# A digital twin approach for simultaneous reconstruction of brain anatomy and dynamics from neural data

**DOI:** 10.1371/journal.pdig.0001445

**Published:** 2026-06-11

**Authors:** Michelangelo Fabbrizzi, Lorenzo Gaetano Amato, Leonardo Martinelli, Jacopo Carpaneto, Emanuele Bartolini, Sara Calderoni, Alessandra Retico, Alberto Arturo Vergani, Alberto Mazzoni

**Affiliations:** 1 The BioRobotics Institute, Sant’Anna School of Advanced Studies, Pisa, Italy; 2 Department of Excellence in Robotics and AI, Sant’Anna School of Advanced Studies, Pisa, Italy; 3 Department of Physics, University of Pisa, Pisa, Italy; 4 Department of Developmental Neuroscience, IRCCS Fondazione Stella Maris, Pisa, Italy; 5 Department of Clinical and Experimental Medicine, University of Pisa, Pisa, Italy; 6 National Institute for Nuclear Physics, Pisa Division, Pisa, Italy; National University of Singapore, SINGAPORE

## Abstract

Brain structure plays a pivotal role in shaping neural dynamics. Current models lack the anatomical and functional resolution needed to integrate whole-brain structure and dynamics within a unified computational framework. Here, we introduce the FEDE (high FidElity Digital brain modEl) pipeline, generating anatomically accurate brain digital twins from imaging data. Combining advanced techniques of finite-element analysis and biophysical modeling, FEDE reconstructs multi-scale brain structure with high spatial resolution, while also replicating whole-brain neural activity. We demonstrated FEDE’s application by creating the first brain digital twin of a toddler with autism spectrum disorder (ASD). Through parameter optimization, FEDE replicated experimental neural activity while reconstructing multi-scale structural features ranging from whole-brain connectivity to synaptic timescales. FEDE estimated possible patient-specific anomalies in synaptic transmission, consistent with ASD pathophysiology. Our pipeline represents a significant leap forward in brain modeling, paving the way for effective applications of digital twins in experimental and clinical settings.

## Author summary

Autism spectrum disorder (ASD) is a common neurodevelopmental condition that affects how people communicate, learn, and interact with others. Despite decades of research, the biological mechanisms underlying ASD remain difficult to pinpoint, partly because we cannot easily study how brain structure and activity interact in patients. In this study, we developed a new approach to bridge that gap based on a digital twin: a detailed computational replica of the patient’s brain. Our method, called FEDE, combines detailed anatomical modeling based on medical images with biophysical simulations of neural activity using finite-element techniques. This allows us to reconstruct both the structure and function of a patient’s brain within the same computational framework. We applied our approach to a young child with ASD, accurately reproducing their recorded brain activity and suggesting possible individualized alterations in synaptic transmission consistent with known ASD mechanisms. By linking anatomy and dynamics in a single model, our approach offers a new avenue for personalized investigation and treatment of brain disorders.

## Introduction

Computational brain models are powerful tools for exploring the structural foundations of neural activity, offering insights into the mechanisms that shape brain dynamics under physiological conditions [1–3]. Beyond fundamental research, these models hold great promise for personalized clinical applications, including disease diagnosis [4] and the evaluation of therapeutic strategies [5,6].

Parallel to advancements in computational modeling, several new MRI preprocessing and postprocessing techniques have been developed, significantly improving our ability to study brain structure with high precision [7–12]. Novel advancements include methods to quantify voxel-wise myelination levels [9,13], crucial for determining the conduction velocity of neural activity across brain regions [14]. Moreover, recent segmentation techniques enable the reconstruction of detailed anatomical properties, such as tissue anisotropy and conductance [15,16], which significantly influence how neural activity is generated and propagated across the brain structure [17].

Despite these separate advancements, no existing model successfully integrates state-of-the-art imaging analysis and computational methods into a unified framework. Each technique requires specific software and packages (see Methods), resulting in a fragmented approach that limits integration. As a result, no existing model can achieve both an accurate reconstruction of brain anatomy and a high-fidelity replication of whole-brain neural dynamics. This limits the potential to develop effective digital twins, virtual models that accurately reconstruct brain anatomy while replicating neural dynamics with the precision required for both in-depth research and clinical applications [18,19].

To address these challenges, we present FEDE (high FidElity Digital brain modEl), a pipeline combining high-resolution biophysical modeling of neural activity with advanced analytic techniques such as reconstruction of local cortical connections, voxel-wise myelination levels and tissue-specific conductance levels. FEDE generates integrative digital brain twins capturing multi-scale anatomy and dynamics.

We validated FEDE by creating the digital brain twin of a toddler (2y 4m old) with autism spectrum disorder (ASD). Developing a digital twin of a toddler presents unique challenges [14], and the multi-factorial anomalies and unclear etiology of ASD [20,21] required FEDE to capture multi-scale anomalies including synaptic imbalance [22,23], abnormal brain growth [24,25] and global and local connectivity alterations [26–28]. However, these challenges and the inter-individual heterogeneity make ASD an ideal candidate for the use of digital twins [18], as both structural and dynamical alterations contribute to the condition’s pathophysiology.

FEDE replicated whole-brain neural activity of the patient (measured via EEG recordings) with high precision, accurately reproducing both temporal and spatial features. Moreover, it identified patient-specific structural alterations that generated the experimentally-observed neural dynamics, with values consistent with ASD pathophysiology.

## Results

**Fig 1:**
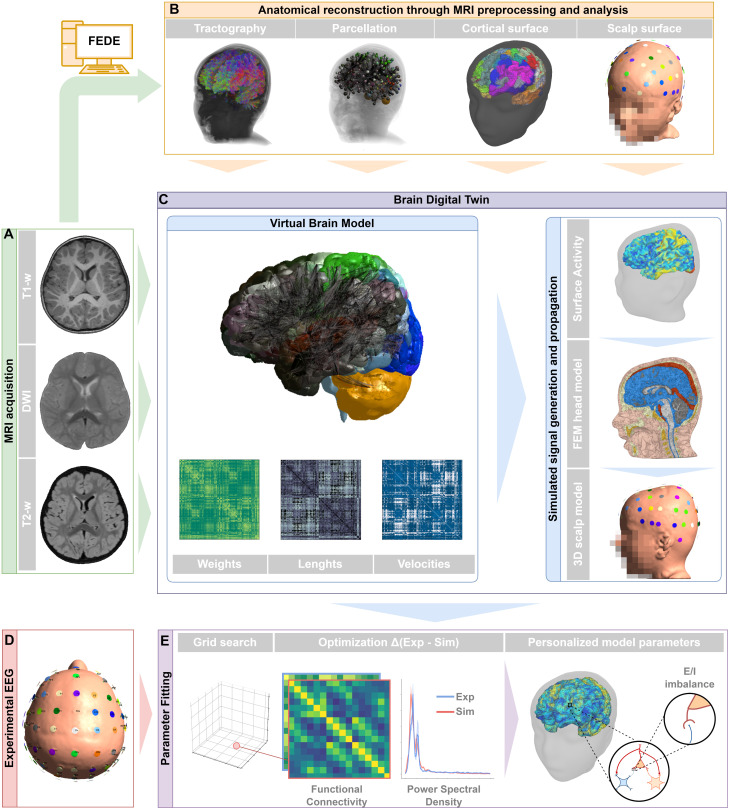
Simultaneous reconstruction of brain anatomy and dynamics from neural data: The FEDE pipeline. **(A):** MRI recordings, including (from top to bottom) T1-w, DWI and T2-w, were used to generate a 3D replica of the patient brain. **(B):** MRI processing steps, from left to right: anatomical constrained tractography analysis, brain regions parcellation and segmentation with reconstruction of cortical surface, reconstruction of biophysical finite element method (FEM) model of the patient’s head, including 3D scalp model with EEG electrodes. **(C):** Left: the parcellation of brain areas defined connective weights, distances and conduction velocity maps for the whole brain structure, which were integrated in a virtual brain model (top, see Methods). Right: neural activity was computed on the high-density cortical surface, the activity was then projected to the scalp of the patient with an anatomically-accurate lead-field matrix, leveraging the FEM model of the patient’s head and considering anisotropy of brain tissues. This allowed for the precise computation of EEG signal from simulated brain activity. **(D):** EEG recordings were acquired during resting-state, extracting features such as power spectral distribution and functional connectivity. **(E):** Parameter optimization through the comparison between experimental and simulated EEG led to the identification of multi-scale structural features underlying patient’s condition.

We developed FEDE, a pipeline for the creation of digital brain twins from comprehensive MRI data (Fig 1A), including T1-weighted (T1-w), T2-weighted (T2-w), and Diffusion-Weighted Imaging (DWI). Briefly, FEDE analyzes MRI data (Fig 1B and Methods) to reconstruct brain anatomy (Fig 1C left and Methods), which is then used as a scaffold to simulate neural activity (Fig 1C right and Methods). Matching simulated signals to experimental recordings (Fig 1E), FEDE reconstructs underlying multi-scale structural features (Fig 1E). FEDE was tested on a toddler (age 2y 4m) with autism spectrum disorder (ASD) (see Methods).

### Personalized conduction velocity map is needed for accurate reconstruction of neural connections

To reconstruct neural connections from MRI data, brain regions were parcellated according to the HCPMMP1 atlas [29]. Connections between regions were computed from DWI data (S1 Fig) and tract lengths were determined from tractography DWI analysis.

**Fig 2:**
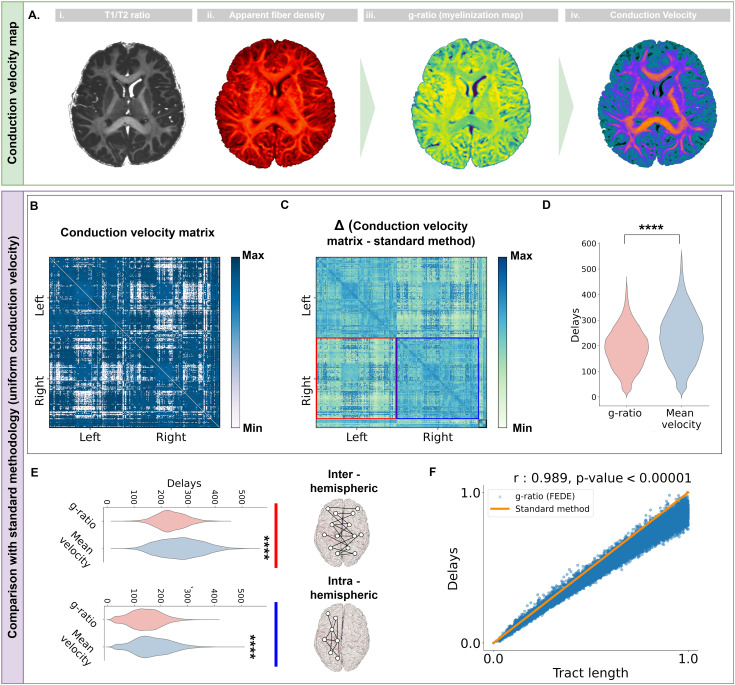
FEDE computes personalized voxel-wise conduction velocity map to precisely reconstruct delays in neural activity transmission. **(A):** Conduction velocity map was computed from the ratio between T1-w and T2-w **(i)**, combined with apparent fiber density (ii) obtained from DWI (see Methods). From the T1/T2 ratio we computed the axonal myelin levels, or g-ratio **(iii)**, which led to the reconstruction of the patient-specific map of conduction velocity of neural activity **(iv)**. Orange indicates high values, while low values of conduction velocities are indicated in blue. **(B):** Conduction velocity matrix ${CV}_{a,b}$, whose entries encode the conduction velocity from region *a* to region ***b*. (C):** Difference between delays computed dividing the tract lengths matrix by the *CV* matrix (TL/CV) and delays computed with standard methodology (i.e., only considering tract lengths). Red (blue) square represents inter-hemispheric (intra-hemispheric) connections, while black box represents subcortical regions. **(D):** Violin plot of delay values computed with the FEDE g-ratio method (red) and standard method (cyan). Delay values computed from a mean conduction velocity value tend to overestimate with high significance actual delay values computed from MRI analysis. **(E):** Violin plot of delay values computed with the two methods, separately for inter-hemispheric connections (top) and intra-hemispheric connections (bottom). Both intra- and inter-hemispheric delays are significantly overestimated with standard methodology (see main text). **(F):** Scatter plot of tract length values and delays computed using the *CV* matrix obtained with FEDE. Orange line represents the expected delays according to standard methodology. Significance notation: **** stands for p < 0.00001.

Alongside standard tractography, FEDE enabled the computation of voxel-wise levels of myelination and conduction velocities. Myelination was computed from T1-w and T2-w imaging combined with apparent fiber density (obtained from DWI [30], see Methods). From this, we determined a conduction velocity map (see Methods), enabling a detailed analysis of signal transmission properties across the brain (Fig 2A, note how lower conduction velocity was observed in the frontal lobe, consistent with the incomplete myelination due to young age).

Combining this voxel-wise map with the selected gray matter atlas, we computed the patient-specific conduction velocity matrix ${CV}_{a,b}$ (Fig 2B, see Methods for details), determining delays in neural transmission between brain areas. We compared the delays computed using FEDE with those derived from the standard methodology, simply proportional to white matter tract lengths without considering myelination. Delays calculated with FEDE were significantly lower than standard values (average ratio between FEDE and standard delays was 0.79 ± 0.05, Mann-Whitney test, U = 1.3e + 11, p < 0.00001, Fig 2C).

Differences in delay times computed with FEDE or with standard methodology were further analyzed by examining inter-hemispheric and intra-hemispheric connections separately (Fig 2E). For inter-hemispheric connections, delays were significantly larger when using the standard methodology (ratio = 0.72 ± 0.04, U = 5e + 9, p < 0.00001). Intra-hemispheric connections also showed statistically significant differences, though less pronounced compared to inter-hemispheric connections (ratio = 0.87 ± 0.05, U = 8e + 9, p < 0.00001).

Delay times computed with FEDE strongly correlated with tract lengths (r = 0.989, p < 0.00001, Fig 2F), but differed from the exact proportionality postulated by standard methodology, being smaller in 96% of the cases ($\chi^{\text{2}}$ test = 7.8e + 5, p < 0.00001). A distribution analysis between FEDE and standard methodology also confirmed statistically significant differences (Kolmogorov-Smirnov test = 0.086, p < 0.00001). These results show that neglecting myelination leads to a consistent overestimation of delay times.

### Biophysical 3D head model enables precise computation of neural activity propagation

**Fig 3:**
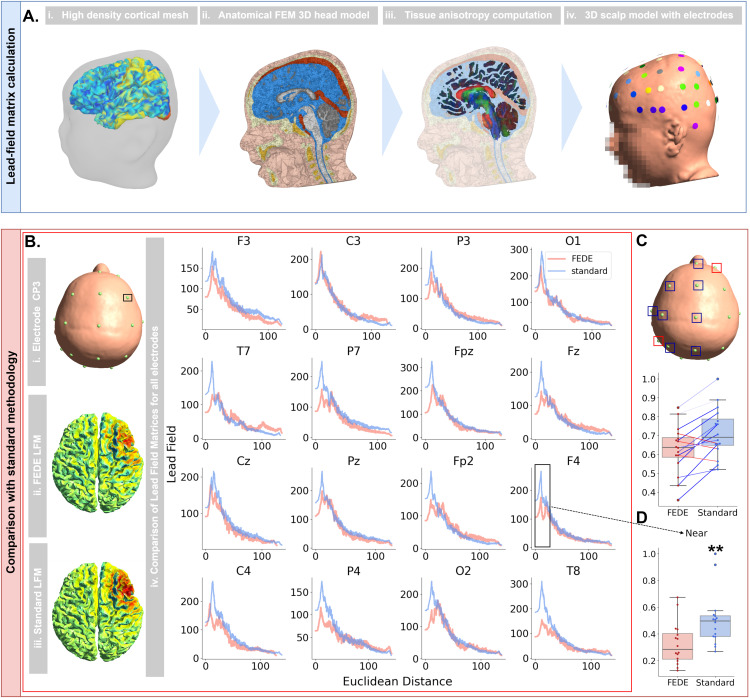
Personalized biophysical 3D head model enables FEDE to precisely locate cortical sources of each EEG electrode. **(A):** From left to right: **(i)** Cortical activity computed on the high-density cortical mesh; (ii) anatomical tissues, each with its own conductance value, were reconstructed with SIMNIBS software, creating a 3D FEM model of the patient’s head, which was combined with tissue anisotropy computed from DWI data **(iii)**. This allowed for the precise propagation of the electric field onto the scalp surface, and thus to the electrode grid **(iv)**. This procedure resulted in the reconstruction of cortical contributions to the activity of each electrode. **(B):** The F4 electrode (i) is plotted as an example, with lead field values computed with FEDE (ii) and standard methodology **(iii)**. **(iv)**: For each EEG channel, lead-field contribution is plotted as a function of the distance to the electrode presented strong differences between FEDE model (salmon) and standard method (light blue). **(C):** Top: Scalp plot highlighting channels presenting statistically significant difference between lead-field values computed with FEDE and with standard methodology. Channels in which the standard methodology led to higher lead-field values (compared with FEDE) are highlighted in blue, while channels in which it led to lower values are highlighted in red. Bottom: Boxplot of average lead-field values for each channel computed with FEDE (salmon) or standard methodology (cyan). Whiskers represent interquartile ranges. **(D):** Boxplot of average lead-field values of near cortical vertices for each channel computed with FEDE (salmon) or standard methodology (cyan). Standard methodology led to significant overestimation of contributions from near vertices. Near vertices are defined (for each channel) as the 20% of closest vertices (in panel B is reported the example of lead-field values of near vertices for the F4 electrode). Notation is the same as in **(C)**. Significance notation: ** stands for p < 0.01, Bonferroni correction.

To model how brain structure shapes the propagation of neural activity, we computed an anatomically-accurate finite element method (FEM) model of the patient’s head using MRI data (Fig 3A). The FEM model accounted for twelve distinct tissue types, with different conductance and anisotropic properties (tissues are reported in S2 Fig). Neural activity was simulated using the Jansen-Rit neural mass model [31] on a high density cortical mesh of 20,484 vertices. The resulting activity was then projected onto the virtual electrodes placed on the scalp surface via a lead-field matrix (LFM, see Methods) derived from computing electric field propagation in the personalized FEM model. Each entry of the matrix ${LFM}_{i,j}$ determines how much cortical vertex *i* contributes to generating the signal at electrode *j*. This procedure ensured that the propagation of simulated neural activity accurately reflected the patient’s unique brain anatomy, which is crucial for modeling electrophysiological signals [32].

For each channel, we compared the distribution of lead-field values computed with FEDE to the values obtained using standard methodology (consisting of a simplified boundary element method of the brain-skull-scalp interfaces, see Methods). Significant differences were found in all channels (Kolmogorov-Smirnov test, p < 0.00001, Fig 3B). We also analyzed channel-wise differences in mean lead-field values, to assess if standard methodology introduced a significant bias in computing cortical contributions to different electrodes. The standard method significantly overestimated lead-field values (p < 0.05) in 9/16 electrodes, while it underestimated lead-field values in 2/16. Notably, 10/11 electrodes that presented statistically relevant differences were located in the central or left portion of the scalp (Fig 3C).

A separate analysis for vertices near the electrode position (defined as the 20% closest vertices for each electrode) showed that standard method significantly overestimated contributions of near vertices to signal generation (0.32 ± 0.08 for FEM vs. 0.50 ± 0.10 for standard method, Fig 3D, U = 57, p = 0.008). On the contrary, differences between the two methods were non-significant for vertices far from the electrodes (defined as the top 20% farthest vertices for each electrode, U = 113, p = 0.58). The interaction between the distance from the electrode and the differences in LFM contribution between standard method and FEDE was mildly confirmed by a two-way ANOVA (f = 3.49, p = 0.066).

### FEDE accurately replicates experimental EEG dynamics across multiple domains

We next measured the accuracy of FEDE in simulating individual brain activity by reproducing the experimental resting-state EEG recordings of a toddler with ASD (see Methods).

**Fig 4:**
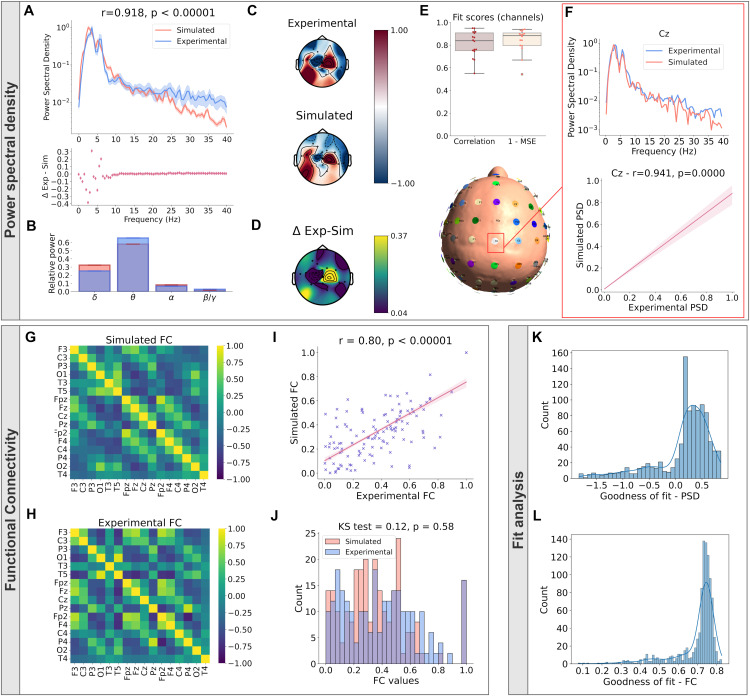
FEDE replicates experimental EEG recordings across multiple domains with high fidelity. **(A):** Patient’s average PSD (top plot, cyan) superimposed to the average PSD reconstructed with FEDE (salmon). Bottom plot shows residual values. **(B):** Relative power in different EEG bands for experimental and simulated recordings. Notation is the same as in **(A)**. **(C):** Topographic plot of scalp EEG activity in the patient and in FEDE, indicating that the model can reproduce the non-trivial topographies encountered in experimental recordings. Signals were normalized between -1 and 1. **(D):** Topographic plot of the (absolute) difference between experimental and simulated EEG activity reported in **(C)**. **(E):** Fit scores of simulated EEG for each channel, reporting correlation with experimental recordings and accuracy (measured as 1 – mean squared error). Each point represents a different EEG channel. Note how correlation was > 0.6 for all channels. **(F):** Cz channel is reported as an example, with PSD (top plot) and regression line between experimental and simulated PSD values (bottom plot). **(G):** FC matrix computed from FEDE’s simulated recordings. **(H):** FC matrix computed from patient’s experimental recordings. **(I):** Linear regression between simulated and experimental FCs, with correlation value reported. FEDE’s FC had a correlation of 0.80 with the experimental one. **(J):** Histogram of experimental and simulated FC values. Kolmogorov-Smirnov test highlighted no significant differences between the two distributions. Notation is the same as in **(A)**. **(K):** Fit values of simulated PSDs for different parameter combinations (only values greater than 0 are reported). **(L):** Fit values of simulated FCs for different parameter combinations (only values greater than 0 are reported). While most combinations of model parameters produced FCs similar to the experimental one, only a few combinations allowed for the correct replication of the PSD.

We determined the combination of model parameters leading to the optimal replication of experimental EEG recordings using functions that determined the similarity between simulated and experimental PSD and FC (fit functions, see Methods). PSD of the simulated EEG displayed high resemblance to the experimental EEG PSD (Fig 4A), as confirmed by linear regression analysis (r = 0.92, $r^{\text{2}}$=0.84, p < 0.00001). We compared simulated and experimental EEG across frequency bands (Fig 4B), finding that the relative power of the digital twin’s PSD correctly replicated the theta and delta dominant rhythm observed in the experimental recording.

The model also reproduced the complex topography observed in experimental recording (Fig 4C and 4D), maintaining consistent correlations with experimental EEG across all channels. This was confirmed by a linear regression analysis between simulated and experimental EEG in each channel, finding an average correlation value of r = 0.81 ± 0.11, and a mean squared error of 0.16 ± 0.10 (Fig 4E, see also S3 Fig, Panel A for channel-wise comparisons between simulated and experimental PSDs). Channel-wise analyses were also conducted for each frequency band, to assess the ability of FEDE in replicating the experimental relative powers in each band and for each channel. No relevant differences between experimental and simulated signals were found (Kolmogorov-Smirnov test p > 0.1, S1 Table, and S3 Fig, Panel B).

FEDE also correctly replicated experimental FC, measured as Pearson correlation between time series of different channels (Fig 4G-H, see Methods). Similar to PSD, simulated and experimental FC were compared using a linear regression analysis, finding high correlation values (Fig 4I, r = 0.80, $r^{\text{2}}$=0.64, p < 0.00001). Most notably, no statistically significant difference was found between distributions of simulated and experimental FC values (Kolmogorov-Smirnov test = 0.13, p = 0.58, Fig 4J). FC replication was also computed with phase slope index (PSI) [33], which is less affected by volume conduction, obtaining similar results (r = 0.53, p < 0.00001) without a dedicated parameter fitting (which would have likely increased the fit value for the PSI metric).

Since both PSD and FC values represent data that are highly intercorrelated, to better quantify the similarity of FEDE-derived metrics with their empirical counterparts, a dependence-aware statistical comparison was conducted between empirical and simulated PSD and FC matrices. This analysis revealed negligible differences for both features, with extremely small effect sizes and non-significant permutation tests (PSD: Cohen’s d = −0.034, p = 0.39; FC: Cohen’s d = 0.025, p = 0.85), indicating that the simulations closely reproduce the empirical distributions.

Of note, despite the generally good performance in replicating experimental EEG features, only a few parameter combinations yielded high similarity with experimental PSD (measured with the PSD fit function, see Methods and Fig 4K), while many combinations reached high similarity with experimental FC (measured with FC fit function, see Fig 4L). This finding probably derives from the dependence of FC values from underlying structural connections, reconstructed with high fidelity by the MRI analytical tools implemented in FEDE.

Next, we evaluated FEDE’s performance in replicating experimental recordings by comparing it to a standard digital twin model. Specifically, we simulated EEG activity using a model with a simplified LFM, disregarding anisotropy and tissue segmentation. Neural activity was computed using a low-density neural mass model [34] without local connectivity effects and assuming a homogeneous conduction velocity (see Methods). The structural connectivity matrix in this standard model was still derived from the FEDE preprocessing pipeline, which incorporated anatomically constrained probabilistic tractography.

**Fig 5:**
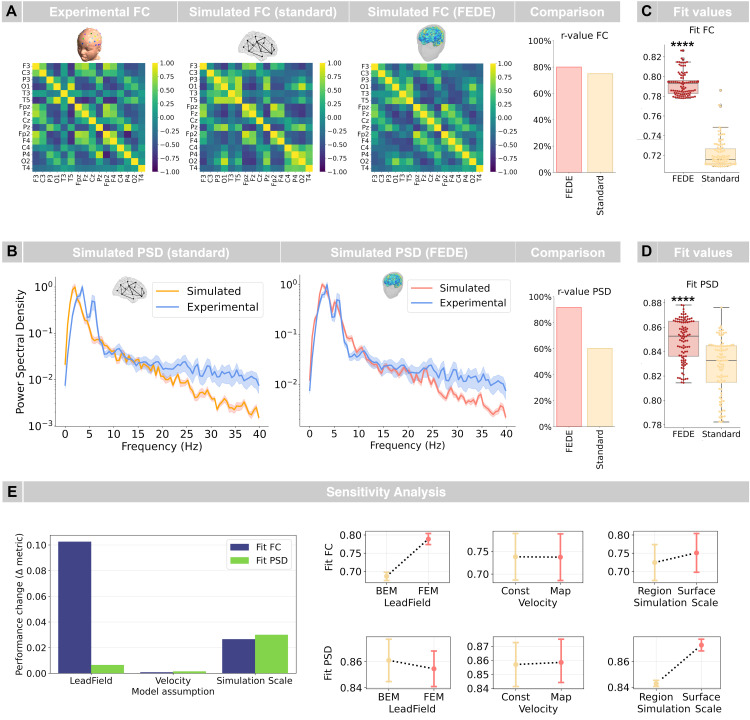
FEDE significantly outperforms standard digital twin models in replicating experimental recordings. **(A):** Comparison of FC matrices obtained from experimental recordings (left), simulated EEG using standard model (middle-left) and simulated EEG using FEDE (middle-right). FC matrices computed from standard methodology showed different patterns with respect to those observed in experimental FC. Standard model led to simulated EEG with a smaller Pearson r-value with experimental FC compared to the value obtained with FEDE (right). **(B):** PSD computed from standard model simulations (left) and from FEDE simulations (middle). The EEG simulated with the standard model presented reduced similarity with experimental recordings, with different dominant frequency and no second peak in low-alpha band. This caused the standard model simulation to have a smaller Pearson r-value with experimental PSD compared to the value obtained with FEDE (right). **(C):** Fit values between experimental and simulated FC for 100 best standard model and FEDE simulations. FEDE presented higher fit values in almost all simulations. **(D):** Fit values between experimental and simulated PSD for 100 best standard model and FEDE simulations. FEDE presented higher fit values with high significance. Significance notation: **** stands for p < 0.00001. **(E):** Sensitivity analysis reporting the relationship between modelling choices and Fit PSD and Fit FC values. Modeling choices include: (i) lead field model (boundary element method, BEM, vs finite element method, FEM), (ii) conduction velocity model (spatially constant vs personalized map), and (iii) simulation scale (region-based vs surface-based simulations). Significance notation: **** stands for p < 0.00001.

We identified the optimal parameters for the standard model and compared simulated results with those obtained using FEDE. In terms of simulated FC, the standard approach resulted in reduced fidelity with respect to FEDE (Fig 5A), with the correlation declining from r = 0.80 to r = 0.75. Regarding PSD, results indicated a more pronounced decrease in fidelity when compared to FEDE, with correlation between experimental and simulated PSDs dropping from r = 0.92 to r = 0.60. Furthermore, this method produced simulated signals with a different dominant frequency than the experimental PSD, also lacking key features such as the secondary peak in the PSD (Fig 5B).

To evaluate the robustness of both methods, we analyzed the similarity between experimental and simulated PSDs and FCs for the top 100 parameter combinations of each method. Similarity was measured using the fit function values of simulated FCs and PSDs (see Methods). In both cases, simulations generated by the FEDE model significantly outperformed those generated by the standard method (Mann-Whitney U test: U = 9969, p < 0.00001 for FC, Fig 5C; U = 7685, p < 0.00001 for PSD, Fig 5D).

Notably, the similarity between experimental and simulated FC remained high even in the simplified model, probably reflecting the high anatomical accuracy of the connectome reconstruction obtained with FEDE. However, the absence of anatomical details in the LFM and in the cortical regions of the standard model significantly decreased the ability to capture finer details of the experimental PSD and FC.

To quantify the relative influence of key modeling assumptions on the performance metrics, we performed an ablation study (Fig 5E) in which we systematically varied the type of lead field model (BEM vs FEM), the formulation of conduction velocity (constant vs subject-specific spatial map), and the simulation scale (region-based vs surface-based). For each combination, we computed the resulting Fit PSD and Fit FC values. We then carried out a sensitivity analysis to determine which assumptions had the largest impact on these metrics. This analysis revealed that the simulation scale (region vs surface) exerted the strongest influence on spectral fit (ΔFit PSD = 0.029975), whereas the lead field model (BEM vs FEM) had the largest impact on functional connectivity fit (ΔFit FC = 0.102500). In contrast, the conduction velocity model (constant vs spatial map) had only negligible effects on both metrics, indicating that Fit PSD and Fit FC are largely insensitive to this assumption.

### FEDE employs Hierarchical Parameter Optimization to reconstruct personalized multi-scale structural features of the patient’s brain

Finding the combination of model parameters that optimally replicate experimental brain activity requires a parameter optimization procedure. To this aim FEDE employed a Hierarchical Parameter Optimization, with biophysical parameters describing multi-scale structural features of the patient’s brain (see Methods for details).

**Fig 6:**
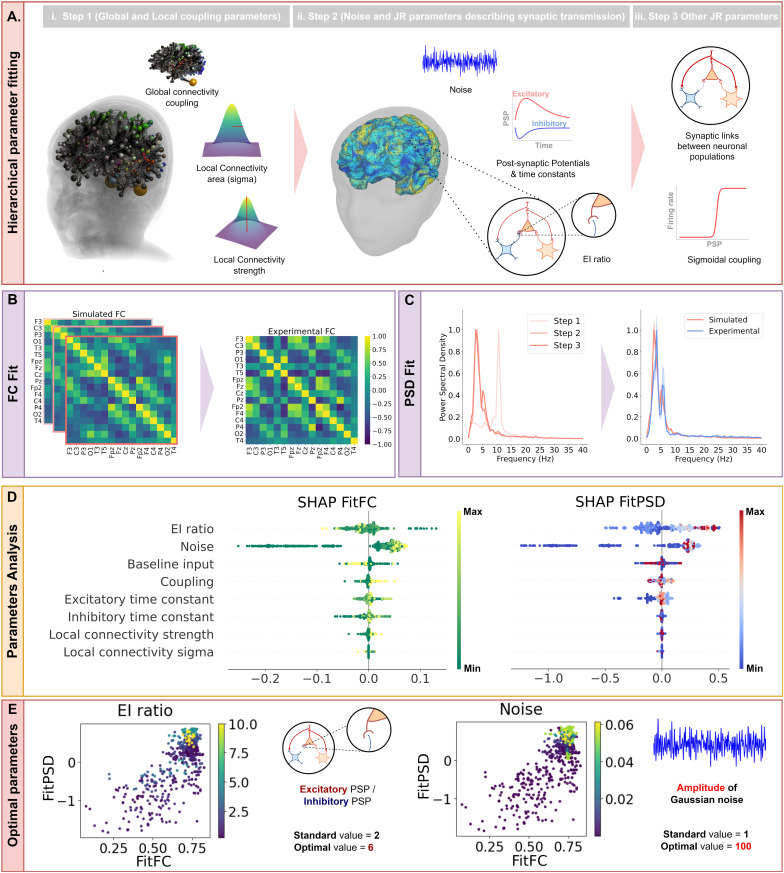
Hierarchical Parameter Optimization enables the reconstruction of multi-scale structural features from experimental recordings. **(A):** The parameter optimization procedure followed three steps. At each step, the procedure identified a combination of model parameters, that was used as starting point for the next step of the parameter optimization procedure. The first step (i) identified values of structural parameters of global and local connectivity, as well as velocity proportionality constant. The second step (ii) identified Jansen-Rit parameters like excitatory and inhibitory post-synaptic potentials (PSP) that dictate the frequency output of the model and mean input firing rate. The third step (iii) identified Jansen-Rit parameters that govern finer features of the simulated output (see for example the second peak of the PSD). The last step focused on the sigmoid transforming membrane potential into firing rate. **(B):** At each step, the fidelity in replicating FC increased, thanks to the identification of more precise parameters. **(C):** Similar increase was also observed for PSD. **(D):** The parameter analysis module was implemented to investigate the importance of each parameter in correctly replicating experimental EEG activity, with statistical tools like SHAP analysis. **(E):** The pipeline enabled the identification of the structural features that more robustly determine EEG activity, that in our patient were aberrant values of the EI ratio and of background noise. FEDE includes tools for the visualization of fit values in relation to model parameters values. In the two scatter plots each dot is a different simulation, higher values of the corresponding parameter (respectively EI ratio and noise) are reported in yellow. See how higher values of both parameters provided high fit values for both PSD and FC. Noise in panel (E) refers to the noise scaling factor (optimal value = 100) multiplied by the excitatory post-synaptic potential over the excitatory characteristic time constant.

In each step of the procedure (Fig 6A), FEDE operates a grid search over a range of candidate parameters, identifying the optimal combination by maximizing the fit functions (see Methods). In the first step, FEDE determines the optimal values of parameters describing connections between brain areas. These parameters include the global connectivity coupling, the area and the strength of the local connectivity gaussian kernel (Step 1 in Fig 6A), and the conduction velocity proportionality constant. The second step comprises the identification of parameters determining the frequency output of the Jansen-Rit model, including the post-synaptic potentials, noise and time constants of excitatory and inhibitory subpopulations, as well as the mean input firing rate. The ratio between inhibitory and excitatory time constants also determines the excitation to inhibition ratio (EI ratio) of the brain. The last step involves the tuning of Jansen-Rit parameters that determine finer effects on output activity, such as the total number of connections in the neural mass, the magnitude of links between single subpopulations, or the sigmoid term transforming membrane potential into firing rate [31]. At each step, the initial point of the grid is determined by the best match identified in the previous step. In our patient, this procedure led to an increase of the precision of both simulated FC (Fig 6B) and PSD (Fig 6C) at each step.

FEDE also incorporates a module for the analysis of fitted parameters, allowing identification of the structural brain parameters playing the most relevant role in the agreement with experimental EEG activity of the patients (see Methods and Fig 6D). A SHAP analysis (see Methods) is implemented to quantitatively identify the model parameters that allow to replicate at best experimental EEG activity. The analysis revealed that only two parameters presented relatively high SHAP values, being EI ratio (0.0033 ± 0.008) and noise (0.0041 ± 0.024), while all other parameters presented values <0.00001.

Since model parameters describe structural features spanning multiple scales, the optimal values identified by FEDE can be compared with physiological values to make hypotheses on underlying patient condition. In our patient, this approach led to the identification of aberrant background noise (whose optimal value was 100 times greater than the standard one [31,35], Fig 6E) and EI ratio (found to be three times greater than the standard healthy value [31], see Fig 6E), which were essential in correctly replicating the experimental EEG activity. Aberrant values of both parameters are consistent with current hypotheses on ASD pathophysiology [22,23,36,37].

## Discussion

Our approach incorporated several advanced analytical techniques into an integrative pipeline. The use of a detailed conduction velocity map derived from MRI data provided a more accurate and nuanced understanding of neural activity transmission delays. It corrected the biases introduced by the traditional approach, ensuring that the physiological variability in conduction velocity across white matter fibers was properly accounted for. In Newman et al. [14], a similar procedure based on the derivation of a conduction velocity map from MRI recordings highlighted significant differences between healthy and ASD subjects. Our approach allowed us not only to reconstruct the conduction velocity maps, but also to assess the role played by this quantity in shaping whole-brain activity.

A critical component of our methodology was the anatomically-accurate computation of the lead-field matrix, which defines the contribution of neural activity from different cortical areas to the signals recorded by each EEG electrode. Our high-resolution cortical model allowed for an accurate calculation of this matrix, minimizing potential errors in source localization. This accuracy was reflected in the detailed comparison of the simulated and experimental EEG data, where the spatial correlations of neural activity (the FC matrix) closely replicated real-life recordings. In current computational brain models, the LFM is derived from an approximated boundary element method based on the compartments between brain, skull and scalp [5,6,38,39]. However, this approach neglects several aspects of brain anatomy, such as the orientation of cortical sources and the presence of several intermediate tissues between sources and electrodes with different conductivity values.

In FEDE, neural activity was computed from local sources on a high density (20,484 vertices) cortical mesh, as lower resolution can prevent the correct replication of experimental neural activity (see S4 Fig). Other works also reported similar or even higher resolutions (e.g., in Wang et al. [6] neural activity was simulated from a cortical mesh of more than 200,000 vertices). However, in these works model inversion is performed in a neural mass model network of reduced dimension and then translated into the high-density mesh. This prevents the model from fitting finer parameters like local connectivity (which are absent in the neural mass model). Our approach includes the parameter optimization procedure directly on the activity computed on the cortical mesh, enabling not only a precise replication and localization of neural activity, but also the reconstruction of fine-scale structural features.

FEDE allowed us to replicate both spatial and time-frequency features of EEG activity. Several works have attempted the replication of PSD [5,38,40] computed from electrophysiological recordings, none reporting the high fidelity obtained with FEDE. The replication of experimental EEG functional connectivity with personalized brain modeling has instead never been attempted before to the best of our knowledge. Several studies focused on the replication of FC matrices deduced from functional MRI (fMRI) recordings [41–45]. Modeling fMRI functional connectivity is more feasible than reproducing functional connections in EEG, as the high spatial resolution of functional MRI allows for precise localization of cortical sources and reduced volume conduction effects, which heavily affect EEG FC metrics [33].

The choice of testing our pipeline with the creation of the first digital brain twin of a toddler patient affected by ASD also holds important implications. Both modelling a toddler brain and capturing the complexities of ASD presented unique challenges yet to be addressed by current models. Acquisition and preprocessing of MRI and EEG data in toddlers are affected by subject restlessness, causing several motion artefacts, while incomplete brain formation requires the use of personalized analytical tools. The realization of a high-fidelity digital twin replica of a toddler with ASD underscores the potential of personalized precision medicine to revolutionize the diagnosis and treatment of neurodevelopmental disorders, paving the way for more effective and individualized therapeutic strategies.

The introduction of a module for parameter analysis led to the identification of structural determinants of experimental EEG features. This is crucial for the translational use of FEDE, as it allows to reconstruct multi-scale structural features from experimental recordings. In the case of our patient, FEDE identified aberrant values of EI ratio and background noise. Both quantities are currently hypothesized to contribute in shaping ASD pathophysiology [22,36]. The ability to infer multi-scale structural features from experimental recordings is particularly valuable in conditions like ASD, which could potentially be the result of multiple concurrent impairments. This approach can also be generalized to a multitude of conditions and recording techniques.

Our study presents several limitations that should be addressed in future research. Firstly, the primary limitation is that our work was conducted on a single patient diagnosed with ASD. While this allowed us to demonstrate the feasibility of creating a digital brain twin in such a complex case, the generalizability of the approach should be tested in further studies. The absence of a control group or additional subjects further restricts our ability to draw definitive conclusions about the broader applicability of the pipeline. This will be key in future activities. Additionally, while complexity of brain development and ASD are unique, and while our approach seems to be promising, it remains uncertain whether it will perform equally well in other populations, such as older children or adults with ASD, or those with other neurological conditions. Future studies with larger and more diverse cohorts are essential for validating our pipeline and assessing its broader utility in personalized medicine. Furthermore, while FEDE introduces several advances in personalized brain modeling, it still represents a simplification of the inherent complexity of the human brain and its intricate interplay of structure and dynamics.

Overall, FEDE represents a significant advancement in brain modeling, providing an effective tool capable of integrating anatomical analytic techniques with high-fidelity biophysical modeling of brain activity. Its possible application to multiple states and conditions opens new avenues for the use of computational brain models in research and clinical settings.

## Materials and methods

### Ethics statement

This study was conducted in accordance with the Declaration of Helsinki and approved by the Regional Ethical Committee of Meyer Hospital (Florence, Italy), number 131/2024 (Approval Date: 19 July 2024). Parents of the subject whose data are analyzed in this manuscript have given written informed consent (as outlined in PLOS consent form) to publish these case details.

### Experimental design

The FEDE pipeline was tested on a pediatric patient with a confirmed diagnosis of ASD. FEDE needs structural data to create the brain digital twin, that we obtained with MRI acquisition. FEDE can also replicate experimental neural activity, reconstructing multi-scale structural features through parameter optimization.

### Subject recruiting, ethical approval and data acquisition

The proband is a male toddler of 2y 4m with a diagnosis of ASD formulated by a multi-disciplinary team according to the Diagnostic and Statistical Manual of Mental Disorders-Fifth Edition [46] criteria, and supported by the administration of the ADOS-2 Toddler Module [47]. The child’s developmental level was measured by an experienced psychologist through the Griffiths Scales of Child Development 3rd Edition [48], and the following scores were obtained: scale A (Foundations of Learning) 99; scale B (Language and Communication) 60; scale C (Eye and Hand Coordination) 105; scale D (Personal–Social–Emotional) 88; scale E (Gross Motor) 105. Moreover, the Childhood Autism Rating Scale second edition (CARS-2) [49] was administered by a qualified evaluator with clinical experience in ASD, and resulted in an overall score of 36, which indicates a level of moderate autism. He underwent both neuroimaging and neurophysiological investigations for clinical purposes at a tertiary care university hospital, the IRCCS Fondazione Stella Maris (Pisa, Italy), to exclude brain anomalies. This study was conducted in accordance with the Declaration of Helsinki and approved by the Regional Ethical Committee of Meyer Hospital (Florence, Italy), number 131/2024. Informed consent for the study was provided by the patient’s legal guardians.

### MRI acquisition

The subject’s data were acquired in a clinical setting at IRCSS Stella Maris in Pisa (Italy). MRI was acquired with a 3T General Electric scanner (Signa HDx, GE-Medical-Systems, Milwaukee, United States). The acquisition protocol was optimized for neurodevelopmental disorders. It included: T1-weighted (T1-w) fast-spoiled gradient echo with voxel size of 1.0mm by 0.4297 mm by 0.4297 mm, TR = 2471.42ms, TE = 3.824ms; 3D T2 fluid-attenuated inversion recovery, two-dimensional (2D) T2-weighted (T2W) fast spin echo with voxel size of 0.799805mm by 0.5 mm by 0.5 mm; Diffusion Weighted Imaging (DWI) with voxel size of 0.9375mm by 0.9375mm by 5.0mm 30 gradient encoding directions and B = 1000s/mm2 TR = 3100ms, TE = 59.6ms.

### Experimental EEG acquisition and pre-processing

EEG data were acquired using the Micromed System Plus Evolution (Micromed, Mogliano Veneto, Italy) with a sampling rate of 256 Hz, utilizing a 21-electrode system adhering to the standard 10–20 layout. The recorded signals were imported into MATLAB’s plugin EEGLAB [50] for further processing. Initially, the resting state epoch was extracted from the continuous EEG recording to capture the subject’s brain activity in a relaxed state without any task-specific stimuli. The EEG data were then filtered in the 1–40 Hz range using a Hamming windowed sinc filter, following an initial high-pass filtering at 1 Hz to remove slow drifts and baseline wander, ensuring the relevant frequency components were retained for analysis.

To enhance the quality of the EEG recordings, the cleanrawdata plugin in EEGLAB was used to identify and remove bad channels and segments. Channels that were silent or flat for more than 5 seconds, exhibited a large amount of noise based on their standard deviation (with a rejection threshold of 4 standard deviations), or had low-frequency signals poorly correlated with nearby channels (using a threshold value of 0.8) were removed. This resulted in a final number of 16 channels selected for further experimental analysis. Additionally, bad portions of the data series were rejected using the Artifact Subspace Reconstruction [51] algorithm for regions exceeding 20 times the standard deviation of the calibration data, and further rejection was based on how many channels within a specified time range exceeded a standard deviation threshold, with a maximum out-of-bound channel percentage set at 25%.

The remaining data were re-referenced using the average reference method [52], averaging the signals across all channels and subtracting the mean signal. Independent Component Analysis (ICA) was then performed using the infomax algorithm to decompose the EEG recordings into independent components, which were classified using the ICLabel plugin [53]. Components identified as artifacts (e.g., eye blinks, muscle activity, or electrical noise) were removed from the dataset, ensuring that only clean portions of the recording were retained for further analysis. This preprocessing ensured a robust foundation for accurate characterization of neural activity and the validity of subsequent analyses.

### FEDE pipeline overview

T1-w, T2-w and DWI acquisitions were processed to create the digital brain model. Due to the clinical data not meeting the mandatory requirements of the ‘TVB Image Processing Pipeline’ — specifically lacking B0 Reverse Phase Encoding [10] (RPE), having only one non-zero B-value, and missing FLASH acquisition [12] — a tailored pipeline was developed. This customized pipeline integrates multiple tools and software packages to achieve the necessary preprocessing and analysis:

- FSL and ANTs: Employed for preprocessing steps such as denoising, unwarping, removal of eddy currents, bias correction, and brain mask generation.
- MRtrix3: Utilized for tractography, constructing the connectome, computing structural connectivity (SC) weights, mean tract lengths and conduction velocity.
- FreeSurfer: Used for anatomical reconstruction, cortical mesh creation, and segmentation/parcellation of the brain.
- SimNIBS: Applied to create the Head Model and the EEG Forward lead-field Matrix.
- MNE (MNE-Python): Implemented to generate inputs for The Virtual Brain (TVB).
- The Virtual Brain: To simulate neural activity.

This integrated approach ensures that the subject-specific data, despite its limitations, can be effectively processed and used as inputs for advanced brain modeling within TVB.

### MRI preprocessing

The MRI preprocessing phase involved several critical steps to prepare the imaging data for further analysis [7,54]. Initially, the DWI data underwent denoising to remove noise artifacts and removal of Gibbs’ ringing artifacts, followed by unwarping to correct for geometric distortions [55]. For susceptibility distortion correction, a synthetic reverse phase–encoding (RPE) image was generated using SynB0-DISCO (https://github.com/MASILab/Synb0-DISCO), following the method described by Schilling et al. [10,56]. This approach uses a deep learning model trained on paired T1-weighted and reversed phase–encoded diffusion images to synthesize a distortion-free b0 image, which can then be used within standard FSL topup/eddy pipelines. SynB0-DISCO was specifically developed to enable susceptibility distortion correction when reversed phase–encoding images are not available and has been validated against acquisitions with real reversed phase–encoding data. The method has been increasingly adopted in diffusion MRI studies where fieldmap or reverse PE data are unavailable.

Eddy current-induced distortions were also corrected [57], and bias correction was applied to mitigate intensity inhomogeneities [7]. A brain mask was generated to isolate the brain from non-brain tissues. Preprocessing steps were performed using FSL [58] and ANTs [59] softwares. A possible different segmentation of subcortical structures (by using Freesurfer Infants [60]) was also tested with the current subject, with no relevant differences in segmentation results. Images were subsequently preprocessed following the preprocessing pipeline outlined in Andy’s Brain Book (Jahn, 2022. doi:10.5281/zenodo.5879293).

### Constrained spherical deconvolution

To characterize the white matter structure, a constrained spherical deconvolution (CSD) was performed [8]. This process included estimating the white matter, cerebrospinal fluid and gray matter response functions for the subject, generating fiber orientation densities [61] (FODs) and normalizing them. Due to the limited diffusion data available (only one gradient value with B = 1000s/mm²), the response functions were generated using MRtrix3Tissue (https://3Tissue.github.io), a fork of MRtrix3 [62]. The latter implements the single-shell three-tissue constrained spherical deconvolution (SS3T-CSD) approach [63]. This method enables the estimation of tissue-specific fiber orientation distributions (FODs) for white matter (WM), grey matter (GM), and cerebrospinal fluid (CSF) even from single-shell diffusion data. The algorithm relies on an iterative estimation of tissue response functions and spatial constraints to separate anisotropic WM signal from isotropic GM and CSF components. SS3T-CSD has been widely adopted in the literature and successfully applied to single-shell datasets (e.g., Newman et al. [64]; Yang et al. [65], Jaroszynski et al. [66]).

Regarding the relatively limited number of diffusion directions [30], preprocessing was performed using the dwifslpreproc pipeline in MRtrix3, which internally calls FSL eddy for correction of motion and eddy-current distortions. In accordance with the FSL recommendations for datasets with fewer than 60 diffusion directions, the option --slm = linear was used during eddy correction, which improves the robustness of the estimation in low-direction acquisitions. The --slm = linear option constrains the eddy-current–induced field to a linear spatial model, reducing the number of parameters to be estimated during eddy correction. This simplification improves the stability and robustness of the correction when diffusion datasets contain a limited number of gradient directions, as in the present acquisition. This approach is also described in the methodological work by Andersson & Sotiropoulos [67] and Andersson et al. [68]. While multi-shell acquisitions with a larger number of directions would provide additional microstructural information, the adopted SS3T-CSD framework has been specifically developed to allow robust estimation of fiber orientation distributions and tractography from single-shell diffusion MRI data.

### Tissue boundaries and coregistration

Following deconvolution, tissue boundaries were created, and the diffusion images were co-registered with the anatomical T1-w images [69]. The boundary interface between gray matter and white matter was identified and used as a seed region for streamline generation through Anatomically Constrained Tractography [11]. This step was performed to ensure that the tractography results were anatomically accurate, providing a robust foundation for subsequent connectivity analysis and brain modeling.

### Probabilistic tractography

Probabilistic tractography [70] was performed using Constrained Spherical Deconvolution [8] coupled with Anatomically Constrained Tractography [11] and Spherical-deconvolution Informed Filtering of Tractograms [71] (SIFT2). Dynamic seeding was also employed to ensure robust streamline generation. The maximum length of fibers was set to 250 mm, following the guidelines of the TVB Image Processing Pipeline [72]. We chose a number of streamlines proportional to the number of regions considered in the parcellation (1000 streamlines for each pair of regions). Specifically, this resulted in a number of 71.631 million streamlines for the adopted HCPMMP1 [29] atlas.

### Anatomical reconstruction

Anatomical reconstruction was conducted using FreeSurfer’s `recon-all` command, which performed cortical mesh creation and segmentation/parcellation using the selected HCPMMP1 atlas. The `-autorecon1` command executed the initial five steps, with tailored inputs to improve subsequent skull extraction. Brainmask was checked and small manual adjustments were performed. After the `-autorecon2`, the white matter mask was checked and slightly edited followed by autorecon2-wm; at last, the `-autorecon3` command completed the remaining steps of the anatomical reconstruction process.

### Structural connectivity weights, mean tract length

We employed MRtrix3 [62] to generate the connectome and calculate structural connectivity (SC) weights, intended as weighted (since SIFT2 was used) number of streamlines connecting two regions, and mean tract length.

### Myelin volume fraction, g-ratio and conduction velocity

We conducted a comprehensive preprocessing and analysis of MRI data to derive conduction and myelinization properties [73], following the methodology outlined in Newman et al. [14]. Initially, the T2-w was co-registered to T1-w.

Brain extraction was performed on the T1-w and T2-w images using the bet function from FSL with specific fractional intensity and gradient threshold settings to generate brain-only images and their masks. The intensity values of these images were then standardized. Minimum and maximum intensity values of T1-w and T2-w images were computed using fslstats, and the images were rescaled to a 0–100 range via fslmaths.

Subsequently, a T1/T2 ratio map was generated. We then extracted “fixels” (fiber population within a voxel) from the normalized wmfod using MRtrix3 fod2fixel and computed the sum of the Apparent Fiber Density (AFD) using fixel2voxel.

Coregistration and regridding were achieved by aligning the fixel-derived apparent fiber density [30] (AFD) sum map with the T1/T2 map using the flirt command, followed by transformation matrix conversion (transformconvert) and regridding (mrtransform, mrgrid).

The myelin volume fraction [9] (MVF) and axon volume fraction (AVF), were derived following the method described by Mohammadi and Callaghan [74], computing MVF/AVF ratios and generating a voxel-wise g-ratio map using fslmaths. The equation for the g-ratio is:


$$\begin{array}{c} \;g\;=\;\frac{d}{D}=\sqrt{\text{1}-\frac{MVF}{MVF\;-AVF\;}}\; \end{array}$$ (1)

where (d) is the axon diameter and (D) is the external myelinated fiber diameter.

This g-ratio map was subsequently upper-thresholded. We further computed the voxel-aggregated conduction velocity (CV) based on the derived g-ratio and AVF using the model by Berman, Filo, and Mezer [13], according to the equation:


$$\begin{array}{c} \;CV=AVF\sqrt{-\;ln(g)}\; \end{array}$$ (2)

The CV map was then coregistered with DWI to align with streamlines from tractography.

Finally, the CV map was sampled along the streamlines of tractography to extract mean velocity per streamline (tcksample) and the CV matrix was then calculated (tck2connectome) from this quantity.

### Cortex surface processing

In order to reduce computational time, the cortical mesh was downsampled from FreeSurfer’s original fsaverage with 327680 triangles to the fsaverage5, which has 20,484 vertices and 40,960 triangles (for comparison, the TVB default cortical mesh [35] uses 16,384 vertices and 32,760 triangles).

### Reconstruction of biophysical 3D head model and calculation of lead-field matrix

The replication of EEG recordings requires projecting neural activity from the high-density cortical mesh to the electrodes on the scalp surface, computing a mathematical quantity called lead-field matrix. The lead-field matrix is an $n_{vertices}\;\times \;n_{electrodes}$ matrix, whose entries encode how the activity of each vertex in the cortical mesh can influence the signal in each EEG electrode. We implemented a pipeline to determine patient-specific lead-field matrix based on patient anatomy. Anatomical tissues between cortex and scalp electrodes were reconstructed with the SimNIBS (Saturnino et al. [75]) software.

In order to calculate the lead-field matrix, we first reconstructed a biophysical 3D head model of the patient with a 12-tissue segmentation using SimNIBS4.1.0. The T1-w and coregistered/regridded T2-w were used as input to charm command together with the Freesurfer anatomical reconstruction folder (the SimNIBS template was coregistered to the T1-w using Freesurfer to find the Transformation matrix used to initialize the affine registration of the SimNIBS template to the subject MR). The DWI image was then imported with the dwi2cond command (based on FSL dtifit), in order to calculate the tensors necessary to compute the anisotropic properties of conductivity for GM and WM (see S5 Fig) using the “Volume Normalized” algorithm as shown by Opitz et al [15]. This approach estimates conductivity tensors from diffusion tensor imaging by assuming a proportional relationship between water diffusion and electrical conductivity in white matter. The method relies on diffusion tensor estimation (FSL dtifit) followed by tensor-to-conductivity conversion as described in the SimNIBS framework. This procedure has been widely used for constructing subject-specific conductivity models for electric field simulations in transcranial stimulation and related modeling studies.

We chose an EEG cap from Neuroelectrics following the 10–10 system [76] and with 64 channels (see for example Fig 1) using electrodes with circular shape (10mm diameter and 4mm of saline gel).

The empirical EEG recordings were acquired using a 21-channel cap, which resulted in 16 channels after preprocessing. For the forward model used in the simulations, a standard 64-channel montage following the international 10–10 system was initially employed to construct the leadfield matrix. This choice provides a denser spatial sampling of the scalp and improves the numerical stability and spatial representation of the forward model.

Because both the empirical montage and the 64-channel template follow the 10–10 coordinate system, the electrode positions corresponding to the recorded channels directly overlap with their counterparts in the 64-channel configuration. Therefore, for the comparison with the empirical EEG signals, only the subset of electrodes corresponding to the 16 recorded channels was retained, while the remaining channels of the 64-channel template were excluded from the analysis.

The electrode configuration, combined with the biophysical 3D head model, the conductivity values shown in S2 Fig, and the reconstructed anisotropic properties, led to the precise computation of lead-field matrix using the TDCSLEADFIELD algorithm.

### The virtual brain format conversion

The next step involved the use of MNE to rearrange the cortical mesh and prepare inputs for The Virtual Brain (TVB). A Python script, derived from `convert2TVB_format.py` [72], was employed to achieve this.

The script computes for each vertex its corresponding region according to the selected atlas, and assigns a flag that determines the vertex’s hemisphere, and another flag indicating whether the vertex is in a cortical or in a non-cortical area. We computed a mid-thickness cortical surface by averaging the white and pial surfaces (in order to have a one-to-one match with the SimNIBS cortex), with a dummy region for non-cortical areas [77]. The script also wrote the lead-field matrix in TVB format, converting the vertices coordinates from Freesurfer system (ras-tkr) to image coordinates (Subject World) saving the cortex file in zipped TVB format (normals, triangles, vertices). Then, it converted the electrode coordinates (again from FS ras-tkr to image coordinates “Subject World”) and wrote the EEG electrodes locations in TVB format, also writing in zipped TVB format the SC weight, Mean Tract Length, Mean Velocity, Region Centres, Average Orientation (mean normal of vertices for a triangle).

As last step, the local connectivity matrix of the cortical surface, providing the weight of the lateral connectivity between cortical columns (assumed to be instantaneous), was computed using a gaussian distribution (where amplitude and sigma were selected by the parameter optimization procedure) applied to the geodesic distance on the mid-thickness cortex. Also, conversion to h5 format was performed to use the visualizer of TVB.

To compare the anatomically-accurate forward solution with current standard methodology, we created the Boundary Element Method (BEM) model of the patient using Brainstorm, importing the result of SimNIBS charm and then considering only the interfaces between brain tissue, skull and scalp. Following standard methodology, default conductivity values (measured as conductance over length, S/m) were assigned to different volumes: 0.465 S/m for brain compartment, 0.008 S/m for skull compartment, and 0.465 S/m for scalp compartment [78]. In the standard BEM-derived lead-field matrix, the ratio between conductivity values is 1:1/80:1 [16], however, it was reduced in order to account for the young age of the subject. The lead-field matrix was computed using OpenMEEG [39]. BEM surfaces and electrodes coordinates were converted from the FreeSurfer system (ras-tkr) to the image coordinate system (ras-scanner), and the lead-field matrix was converted into TVB format with the same procedure adopted for the anatomically-accurate one.

### Neural activity simulation

SC data, conduction velocity, region mapping, cortical vertices, electrode positions and local connectivity were imported into TVB to perform the simulations (h5 files were created for import and visualization purposes in TVB GUI). Activity on cortical vertices was simulated using the Jansen-Rit model [31]. The Jansen-Rit neural mass model was chosen for its capacity of replicating EEG recordings [31], but several different models can be implemented to replicate multi-modal acquisitions of neural activity. Subcortical structures were minimally segmented and modeled using the same Jansen-Rit model as the cortex. The simulation of cortical surface activity allows the modeling and analysis of both local and global connectivity [35], as both are reported to be aberrant in ASD [26,28,79,80]. An anatomically-accurate forward solution was used to compute virtual EEG from simulated activity [81]. As already mentioned, the local connectivity was computed from geodesic brain distance by using a gaussian kernel, whose extension and strength was determined by parameter optimization. Model parameters are reported in S2 Table.

### Parameter optimization with experimental EEG recording

To optimize model parameters, a hierarchical grid search procedure was implemented, comparing simulated and experimental recordings at each step (see Results). Parameters to optimize included white matter speed, SC weights scaling, sigmoidal Jansen-Rit coupling, local connectivity, and Jansen-Rit model parameters. For each combination of parameters, a simulated EEG was generated, with a total of 1480 simulations.

Simulated signals were fitted to experimental recordings, based on fit functions that computed the similarity between simulated and experimental functional connectivity and power spectral densities. The two fit functions are:


$$\begin{array}{c} \;Fit\;FC=Corr\left({FC}_{exp}\;,\;{FC}_{sim}\right)\; \end{array}$$ (3)

$$\begin{array}{c} \;Fit\;PSD=\text{1}-MAE[{log}_{\text{1}\text{0}}\left({PSD}_{\text{exp}})-{log}_{\text{1}\text{0}}\left({PSD}_{\text{sim}}\right)\right]\; \end{array}$$ (4)

Where Corr is the standard Pearson correlation. In the ${Fit}_{PSD}\;$ function, the PSD was truncated at 20 Hz (this was done to improve the efficacy of the loss function in locating the best fits), MAE is the Mean Absolute Error as implemented by sklearn.metrics, PSD values were averaged across channels. The best match between simulated and actual EEG was computed using these metrics as the combination of model parameters that maximized the $\sqrt{{FitPSD}^{\text{2}}+\;{Fit\;FC}^{\text{2}}}$ sum.

### EEG features: Power spectral distribution

We computed the power spectral distribution (PSD) using Welch’s method, dividing the signal into overlapping segments, applying windowing, and taking the discrete Fourier transform to obtain power spectrum estimates. These estimates were averaged to reduce variance, producing a binned array of power spectral densities for each frequency.

Oscillations in neural data are embedded within aperiodic activity, typically following a 1/f distribution. Traditionally, this aperiodic component was disregarded or treated as noise. However, variations in aperiodic activity are now recognized as potential biological indicators for disease, aging, and development. Therefore, we parametrized PSDs into periodic and aperiodic components using the FOOOF (fitting oscillations and one-over f) algorithm [82].

FOOOF models the PSD as a combination of 1/f activity *L* and *N* periodic components $G_{n}$:


$$\begin{array}{c} \text{log}\left(PSD(f)\right)=L+\;\sum {\mathrm{\backslash nolimits}}_{n}G_{n} \end{array}$$ (5)

With periodic components $G_{n}$ modeled with Gaussians and the aperiodic component *L* is modeled as:


$$\begin{array}{c} \;L(f)=b-\text{log}\left(k+f^{\alpha }\right)\; \end{array}$$ (6)

where *b* is the broadband offset, *α* is the exponent, and *k* is the knee parameter.

FOOOF involves initial fitting of the aperiodic component, detrending the spectrum, detecting and fitting periodic components iteratively, and combining these fits to create the model, computing goodness-of-fit scores. If the knee parameter k=0, aperiodic exponents are more comparable and interpretable, serving as potential biomarkers.

### EEG features: Functional connectivity

Functional connectivity was computed with standard Pearson correlation, considering only significant (p < 0.05, Bonferroni correction) values. The choice of the Pearson correlation metric was motivated by the limited number of channels, reducing volume conduction effects [33]. Furthermore, volume conduction is heavily determined by the anatomical structure [83], meaning that the correct replication of Pearson correlation requires the reconstruction of both the neural activity and the underlying anatomy at once. Nonetheless, other FC metrics were also tested, computing Phase Slope Index in both empirical and simulated data, with similar results with respect to those obtained for Pearson correlation.

### Ablation study and sensitivity analysis

To quantify the influence of key modeling assumptions on model performance, we conducted an ablation study in which three methodological components were systematically varied: (i) the lead field formulation (boundary element method, BEM, vs finite element method, FEM), (ii) the conduction velocity model (spatially constant vs personalized map), and (iii) the simulation scale (region-based vs surface-based simulations). All possible combinations of these assumptions were evaluated, resulting in eight simulation configurations. For each configuration, we computed two performance metrics quantifying the agreement between simulated and empirical data: (Fit PSD and Fit FC). To assess the relative impact of each modeling assumption, we performed a sensitivity analysis by computing, for each factor, the absolute difference between the average metric values obtained across its levels (e.g., FEM vs BEM). This quantity (Δ metric) provides a direct estimate of how strongly each modeling choice influences the resulting model fit. Full Δ metrics are reported in S3 Table.

### Statistical analysis

Statistical analyses were performed using Python packages, including SciPy and Statsmodels. Group differences in average values were assessed using the Mann-Whitney U test, while distribution differences were evaluated with the Kolmogorov-Smirnov test. Bonferroni correction was applied to account for multiple comparisons. Additionally, SHAP analysis was conducted using the Scikit-learn (sklearn) library to assess parameter importance in the parameter optimization routine. To compare empirical and simulated data, we also performed dependence-aware statistical comparisons between simulated and empirical measures. For functional connectivity matrices, analyses were restricted to the upper triangular part of the matrices to avoid redundancy due to symmetry. Statistical differences were assessed using non-parametric permutation tests, which do not assume independence between observations. In addition to p-values, we quantified the magnitude of differences using Cohen’s d effect sizes.

## Supporting information

S1 Fig Structural connectivity matrix. Structural Connectivity weights between brain regions, reported in logarithmic scale (log_10_(1 + weight)). Structural connectivity matrix is derived from tractography analysis combined with gray matter parcellations. Connections are arranged according to hemispheres subdivisions, while subcortical regions are reported in the bottom and right part of the matrix.
(TIF)

S2 Fig 3D FEM model of patients’ head with tissue and electrodes conductances. Values can be consulted in https://simnibs.github.io/simnibs/build/html/documentation/conductivity.html, alongside references from which the single conductance values are deduced.
(TIF)

S3 Fig Channel by channel comparison of experimental and simulated PSDs. **(A):** Single channels PSD from experimental and simulated signals. **(B):** Violin plot of relative EEG band power of channels. Distribution did not present statistically relevant differences between experimental and simulated signals.
(TIF)

S4 Fig Comparison between simulated PSDs and FCs with region and surface-based simulation. Model parameters selected through parameter exploration were utilized in a region-based simulation, using as scaffold the 379-regions HCPMMP1 atlas employed for gray matter parcellation. Results were compared with both the experimental values and with surface-based simulations. **(A):** PSD computed from region-based simulations fails to capture finer details of the experimental PSD, such as the second peak in low-alpha band. **(B):** FC matrices computed from region-based simulations show a more stereotypical differentiations between highly and low functionally connected electrode. **(C):** r-regression coefficient between experimental and simulated PSD is higher for surface-based analysis. **(D):** Similarly, r-coefficient between experimental and simulated FC is higher for surface-based analysis.
(TIF)

S5 Fig Determination of structural tissue anisotropy. The DWI image was used as input to the *dwi2cond* command (which is based on FSL *dtifit* algorithm), to calculate the tensors necessary to calculate the anisotropic properties of conductivity for GM and WM using the “Volume Normalized” algorithm (see main text).
(TIF)

S1 Table Statistical analysis of differences in PSD relative power between simulated and experimental EEG channels. Kolmogorov-Smirnov test was used, highlighting no statistical differences between the two distributions.
(DOCX)

S2 Table Optimal combinations of model parameters: Parameters of the Jansen-Rit neural mass model are reported first, and separated from the parameters of the brain model by a horizontal line. (DOCX)

S3 Table Comparison between simulated PSDs and FCs depending on simulation features. Quality of fit for functional connectivity (Fit FC, fifth column) and PSD (Fit PSD, sixth column) using simulations with finite or boundary element (FEM or BEM) lead field matrix (LFM, second column), Constant or map-based Conduction Velocity (CV, third column), and region based vs surface based simulation scale (fourth column). Simulations have been made using the optimal combination of model parameter (see S2 Table).
(DOCX)

S1 Data EEG features (Power Spectral Density and Functional Connectivity) and MRI-derived Structural Connectivity matrices (connective weights, tract lengths and conduction velocities) of the participant. (ZIP)
