# Supplementary figures and images for: A digital twin approach for simultaneous reconstruction of brain anatomy and dynamics from neural data
Source: PLOS Digit Health. 2026 Jun 11;5(6):e0001445. doi: 10.1371/journal.pdig.0001445 (PMC13258024; doi:10.1371/journal.pdig.0001445)

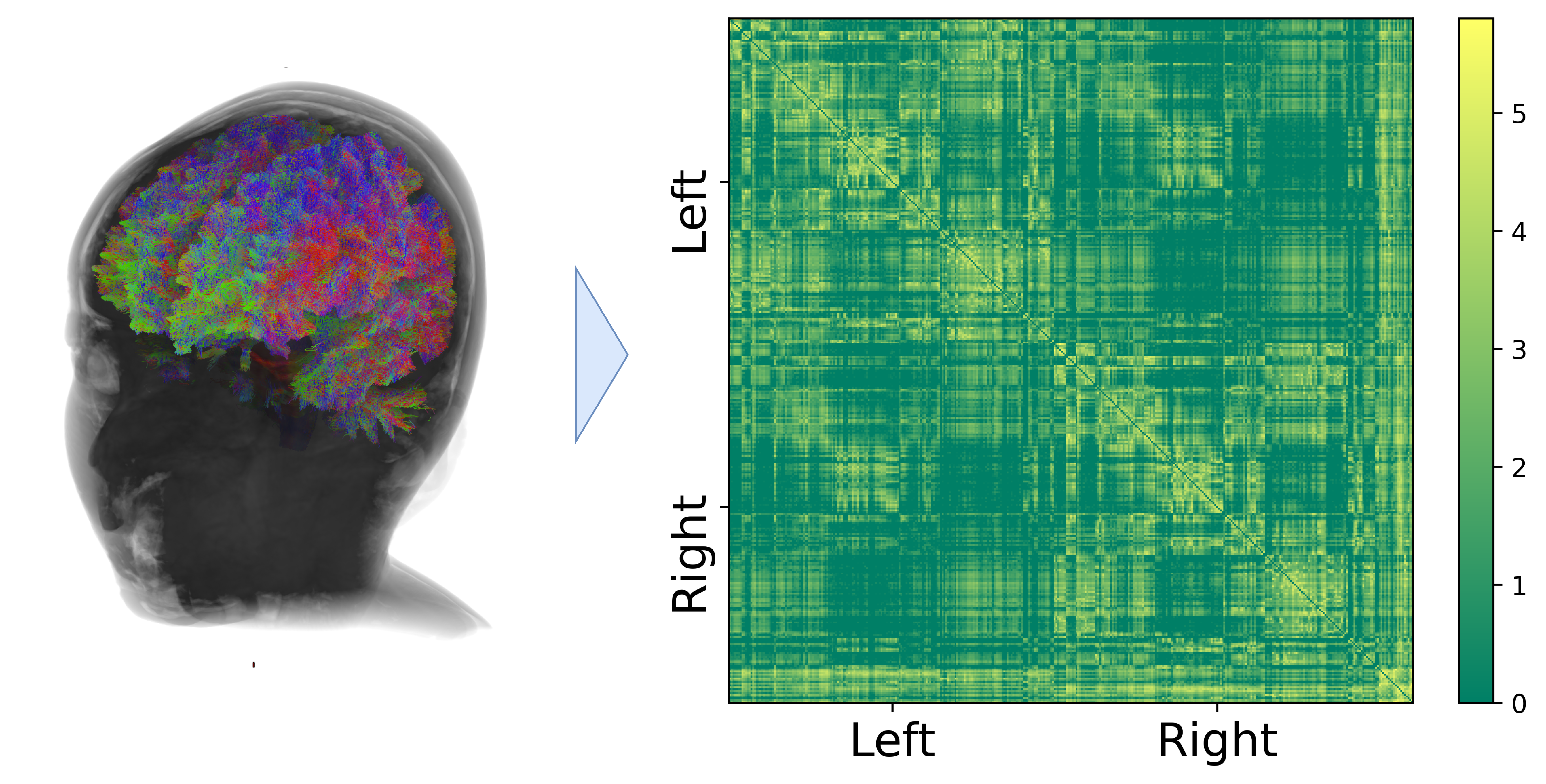

Supplement: S1 Fig — Structural Connectivity weights between brain regions, reported in logarithmic scale (log10(1 + weight)). Structural connectivity matrix is derived from tractography analysis combined with gray matter parcellations. Connections are arranged according to hemispheres subdivisions, while subcortical regions are reported in the bottom and right part of the matrix. (TIF) [file pdig.0001445.s001.tif]

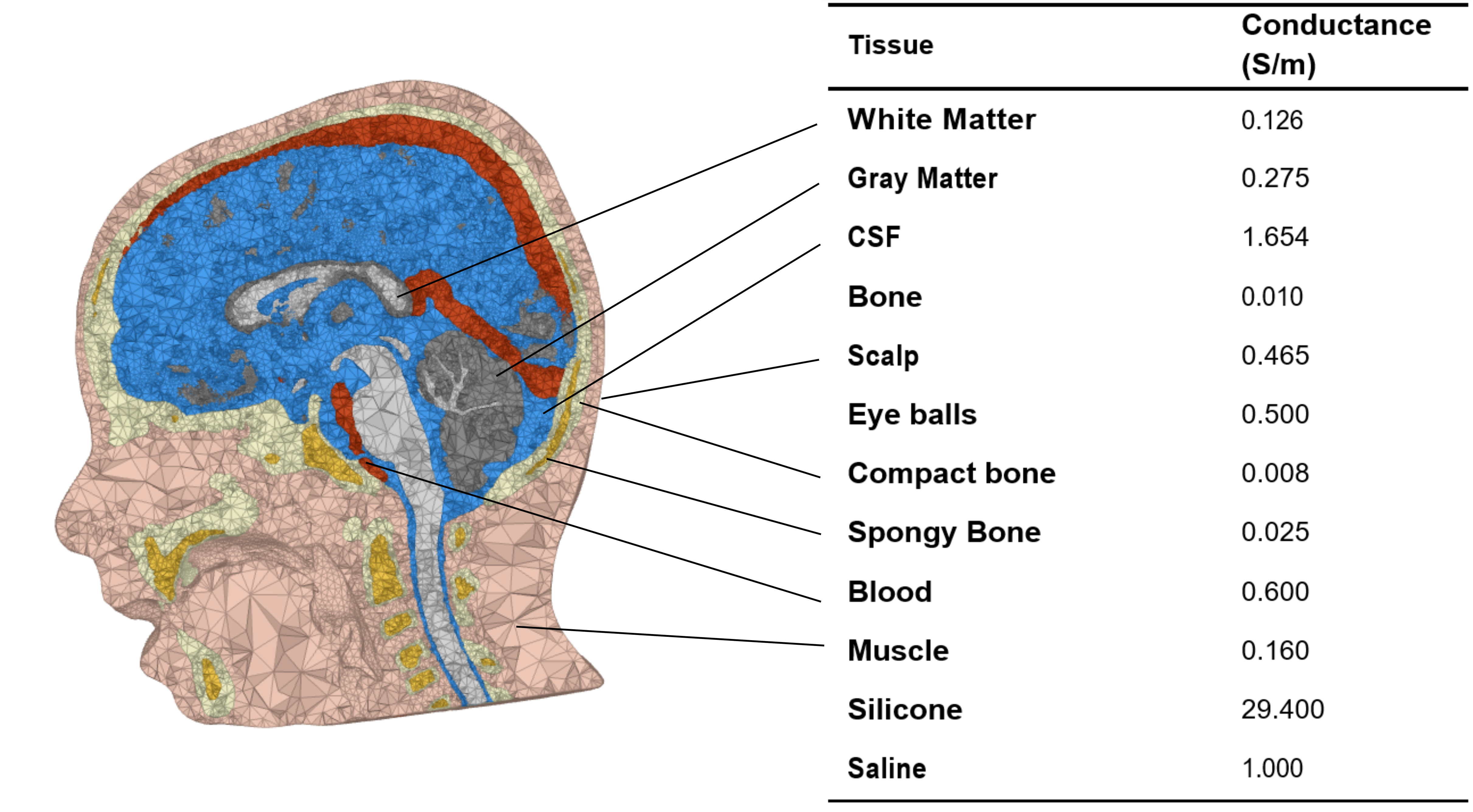

Supplement: S2 Fig — Values can be consulted in https://simnibs.github.io/simnibs/build/html/documentation/conductivity.html, alongside references from which the single conductance values are deduced. (TIF) [file pdig.0001445.s002.tif]

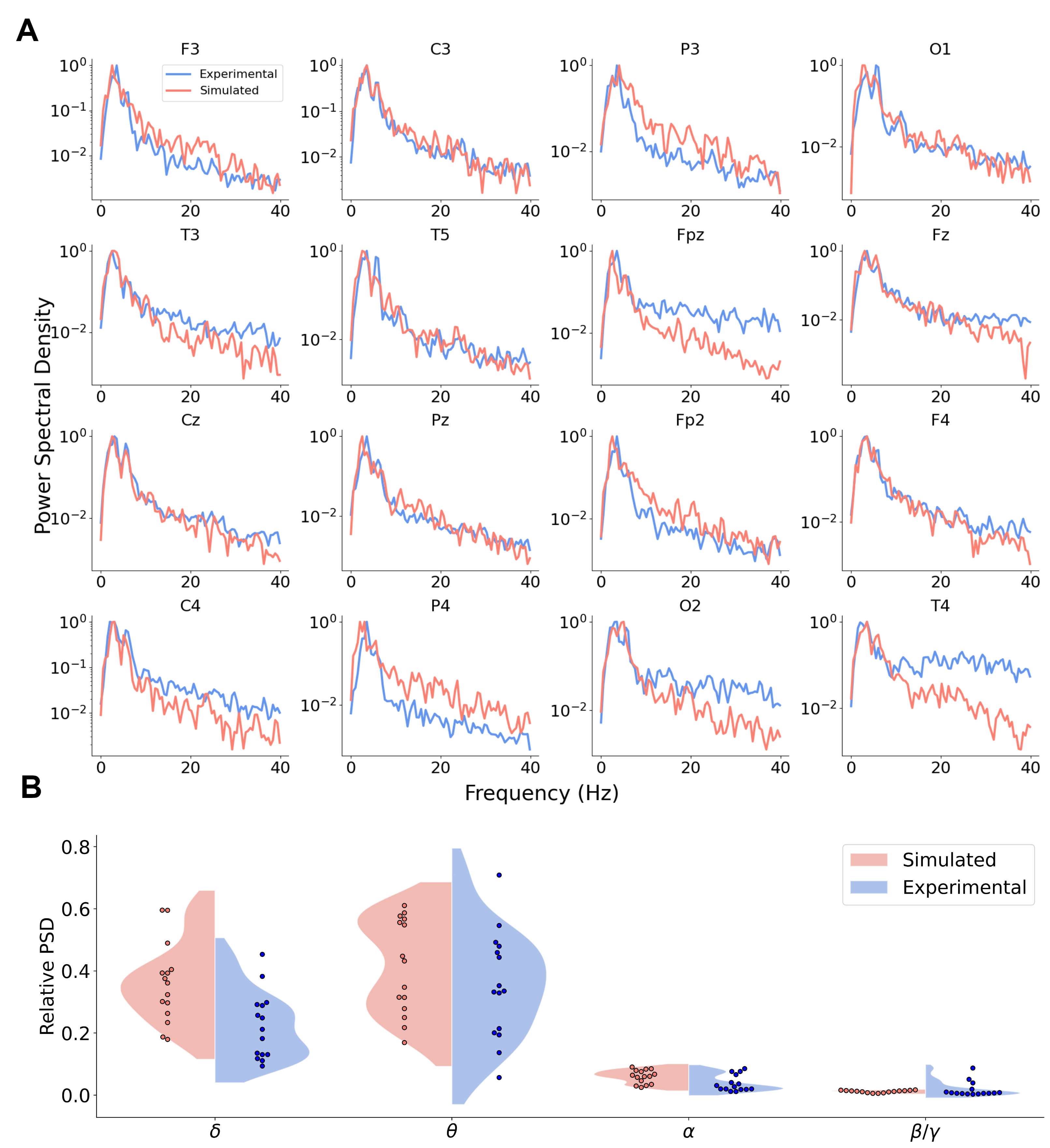

Supplement: S3 Fig — (A): Single channels PSD from experimental and simulated signals. (B): Violin plot of relative EEG band power of channels. Distribution did not present statistically relevant differences between experimental and simulated signals. (TIF) [file pdig.0001445.s003.tif]

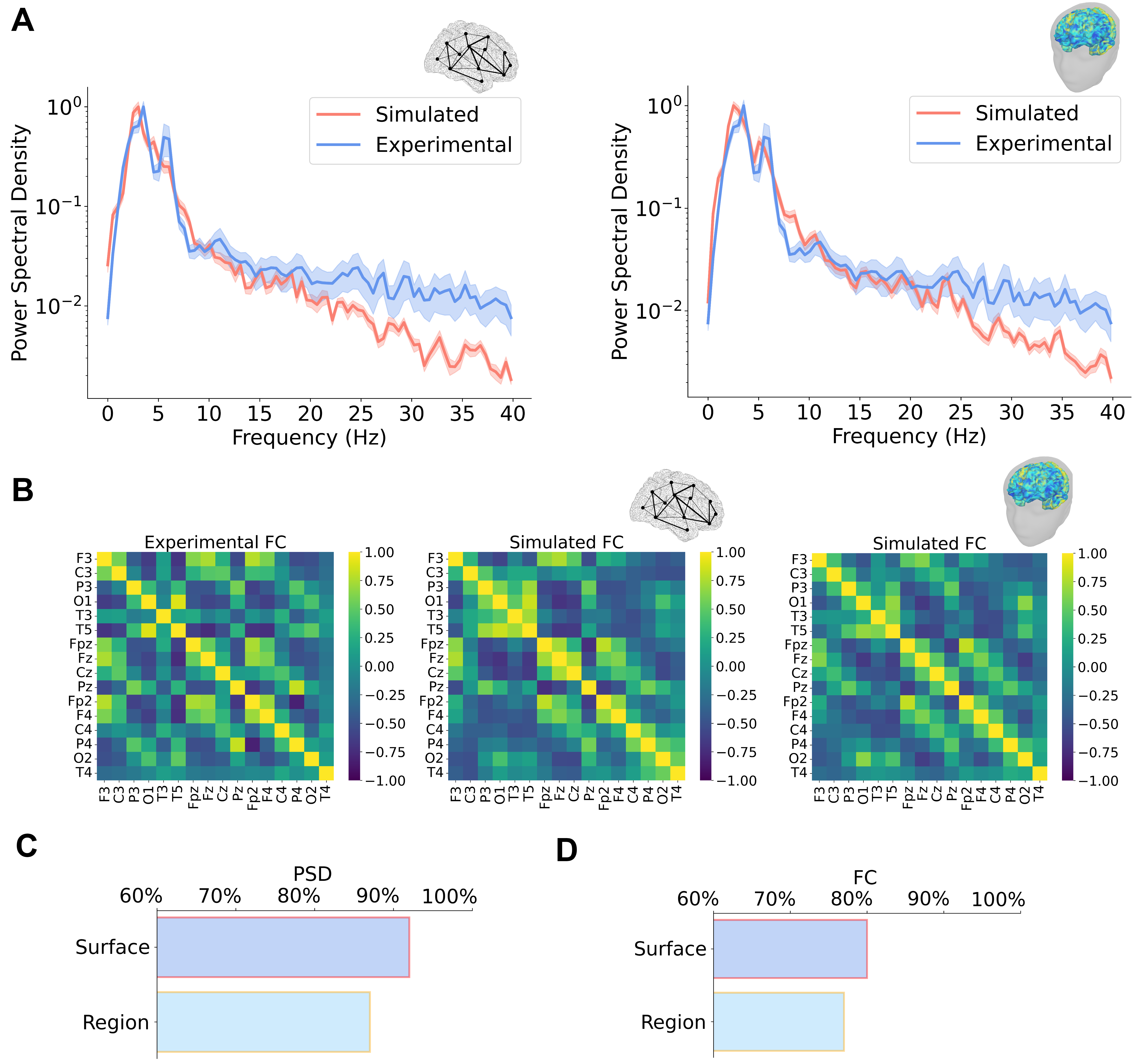

Supplement: S4 Fig — Model parameters selected through parameter exploration were utilized in a region-based simulation, using as scaffold the 379-regions HCPMMP1 atlas employed for gray matter parcellation. Results were compared with both the experimental values and with surface-based simulations. (A): PSD computed from region-based simulations fails to capture finer details of the experimental PSD, such as the second peak in low-alpha band. (B): FC matrices computed from region-based simulations show a more stereotypical differentiations between highly and low functionally connected electrode. (C): r-regression coefficient between experimental and simulated PSD is higher for surface-based analysis. (D): Similarly, r-coefficient between experimental and simulated FC is higher for surface-based analysis. (TIF) [file pdig.0001445.s004.tif]

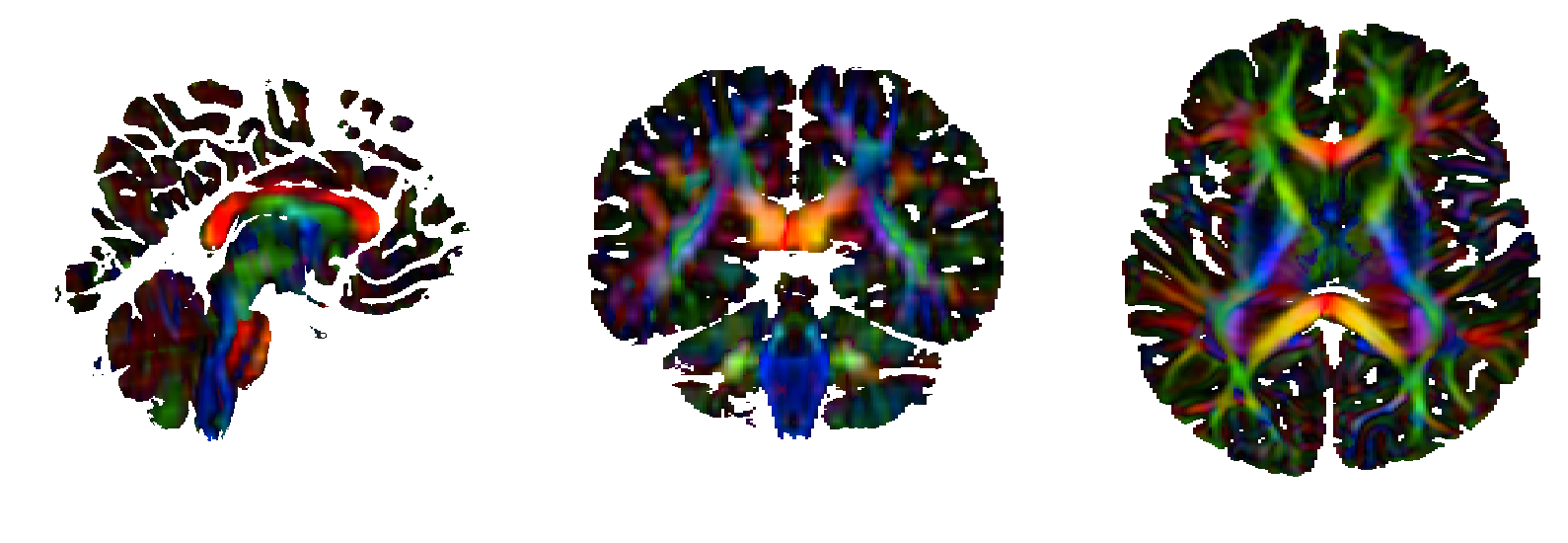

Supplement: S5 Fig — The DWI image was used as input to the dwi2cond command (which is based on FSL dtifit algorithm), to calculate the tensors necessary to calculate the anisotropic properties of conductivity for GM and WM using the “Volume Normalized” algorithm (see main text). (TIF) [file pdig.0001445.s005.tif]
