# Supplementary material for: A digital twin approach for simultaneous reconstruction of brain anatomy and dynamics from neural data
Source: PLOS Digit Health. 2026 Jun 11;5(6):e0001445. doi: 10.1371/journal.pdig.0001445 (PMC13258024; doi:10.1371/journal.pdig.0001445)
Supplement: S1 Table — Kolmogorov-Smirnov test was used, highlighting no statistical differences between the two distributions. (DOCX) [file pdig.0001445.s006.docx]

|  | **KS test** | **p-value** |
| --- | --- | --- |
| **Delta** | 0.53 | 0.10 |
| **Theta** | 0.33 | 0.38 |
| **Alpha** | 0.46 | 0.30 |
| **Beta+Gamma** | 0.46 | 0.30 |

**Table S1.**

**Statistical analysis of differences in PSD relative power between simulated and experimental EEG channels.** Kolmogorov-Smirnov test was used, highlighting no statistical differences between the two distributions.
