# Supplementary material for: A digital twin approach for simultaneous reconstruction of brain anatomy and dynamics from neural data
Source: PLOS Digit Health. 2026 Jun 11;5(6):e0001445. doi: 10.1371/journal.pdig.0001445 (PMC13258024; doi:10.1371/journal.pdig.0001445)
Supplement: S2 Table — (DOCX) [file pdig.0001445.s007.docx]

| **P****arameter** | **Description** | **Measure unit** | **Value** |
| --- | --- | --- | --- |
| *A* | Maximum Excitatory | mV | 3.50 |
|  | post-synaptic potential |  |  |
| *B* | Maximum Inhibitory | mV | 12.0 |
|  | post-synaptic potential |  |  |
| *τe* | Excitatory time constant | ms | 8.3 |
| *τi* | Inhibitory time constant | ms | 50 |
| *v*0 | Voltage threshold for which a | *mV* | 6.0 |
|  | 50 % firing rate is achieved |  |  |
| *νmax* | Maximum firing rate of | s−1 | 0.0025 |
|  | neural population |  |  |
| *r* | Steepness of sigmoidal | mV−1 | 0.56 |
|  | transfer function |  |  |
| *C*1 | Average probability of synaptic contacts | pure number | 1.2 |
|  | in the feedback excitatory loop |  |  |
| *C*2 | Average probability of synaptic contacts | pure number | 0.8 |
|  | in the slow feedback excitatory loop |  |  |
| *C*3 | Average probability of synaptic contacts | pure number | 0.25 |
|  | in the feedback inhibitory loop |  |  |
| *C*4 | Average probability of synaptic contacts | pure number | 0.3 |
|  | in the slow feedback inhibitory loop |  |  |
| *η* | Mean input noise | s−1 | 0.088 |
| *µ* | Mean input firing rate | s−1 | 0.12 |
| *J* | Average internal connections | pure number | 150 |
| WM speed | Multiplicative constant to the conduction velocity matrix | pure number | 30 |
| WM coupling (a) | Multiplicative constant to the structural connectivity matrix (normalized with nr of generated streamlines during tractography) | pure number | 1.86*10 ^-5^ |
| WM coupling (cmin) | Minimum of the sigmoid function | pure number | 0.0 |
| WM coupling (cmax) | Maximum of the sigmoid function | pure number | 0.005 |
| WM coupling midpoint | Midpoint of the linear portion of the sigmoid |  | 3.12 |
| WM coupling (r) | Steepness of the sigmoidal transformation | pure number | 0.28 |
| LC strength | Strength of local connections | pure number | 0.1 |
| LC sigma | Area of influence of local connections | pure number | 0.5 |

**Table S2.**

**Optimal combinations of model parameters:** Parameters of the Jansen-Rit neural mass model are reported first, and separated from the parameters of the brain model by a horizontal line.
