# Supplementary material for: A digital twin approach for simultaneous reconstruction of brain anatomy and dynamics from neural data
Source: PLOS Digit Health. 2026 Jun 11;5(6):e0001445. doi: 10.1371/journal.pdig.0001445 (PMC13258024; doi:10.1371/journal.pdig.0001445)
Supplement: S3 Table — Quality of fit for functional connectivity (Fit FC, fifth column) and PSD (Fit PSD, sixth column) using simulations with finite or boundary element (FEM or BEM) lead field matrix (LFM, second column), Constant or map-based Conduction Velocity (CV, third column), and region based vs surface based simulation scale (fourth column). Simulations have been made using the optimal combination of model parameter (see S2 Table). (DOCX) [file pdig.0001445.s008.docx]

| **Variant** | **LFM Model** | **CV Model** | **Simulation Scale** | **Fit FC** | **Fit PSD** |
| --- | --- | --- | --- | --- | --- |
| 01 | BEM | Const | Region | 0.676 | 0.8429 |
| 02 | FEM | Const | Region | 0.7742 | 0.8398 |
| 03 | BEM | Map | Region | 0.6741 | 0.8465 |
| 04 | FEM | Map | Region | 0.7729 | 0.8419 |
| 05 | BEM | Const | Surface | 0.6975 | 0.8773 |
| 06 | FEM | Const | Surface | 0.8041 | 0.8681 |
| 07 | BEM | Map | Surface | 0.6977 | 0.8773 |
| 08 | FEM | Map | Surface | 0.8041 | 0.8683 |

**Table S3.**

**Comparison between simulated PSDs and FCs depending on simulation features.** Quality of fit for functional connectivity (Fit FC, fifth column) and PDS (Fit PSD, sixth column) using simulations with finite or boundary element (FEM or BEM) lead field matrix (LFM, second column), Constant or map-based Conduction Velocity (CV, third column), and region based vs surface based simulation scale (fourth column). Simulations have been made using the optimal combination of model parameter (see Table S2).
