# Supplementary material for: A digital twin approach for simultaneous reconstruction of brain anatomy and dynamics from neural data
Source: PLOS Digit Health. 2026 Jun 11;5(6):e0001445. doi: 10.1371/journal.pdig.0001445 (PMC13258024; doi:10.1371/journal.pdig.0001445)
Supplement: Attachment — Submitted filename: Response_Letter.docx [file pdig.0001445.s011.docx]

Dear Dr. Amato,

Thank you for submitting your manuscript to PLOS Digital Health. After careful consideration, we feel that it has merit but does not fully meet PLOS Digital Health's publication criteria as it currently stands. Therefore, we invite you to submit a revised version of the manuscript that addresses the points raised during the review process.

Please submit your revised manuscript by May 02 2026 11:59PM. If you will need more time than this to complete your revisions, please reply to this message or contact the journal office at digitalhealth@plos.org. When you're ready to submit your revision, log on to https://www.editorialmanager.com/pdig/ and select the 'Submissions Needing Revision' folder to locate your manuscript file.

Please include the following items when submitting your revised manuscript:

* A letter that responds to each point raised by the editor and reviewer(s). You should upload this letter as a separate file labeled 'Response to Reviewers'. This file does not need to include responses to any formatting updates and technical items listed in the 'Journal Requirements' section below.

* A marked-up copy of your manuscript that highlights changes made to the original version. You should upload this as a separate file labeled 'Revised Manuscript with Track Changes'.

* An unmarked version of your revised paper without tracked changes. You should upload this as a separate file labeled 'Manuscript'.

If you would like to make changes to your financial disclosure, competing interests statement, or data availability statement, please make these updates within the submission form at the time of resubmission. Guidelines for resubmitting your figure files are available below the reviewer comments at the end of this letter

We look forward to receiving your revised manuscript.

Kind regards,

Leo Anthony Celi

We are thankful to the Editor for the kind comments and this occasion to improve our work. Below you will find a point-by-point reply to the comments of the reviewers. In particular, comments from Reviewers 2 and 3 led to a deep revision of the Methods section to improve the clarity of the manuscript and ensure a proper reproducibility of our approach, and to a novel sensitivity analysis significantly improving the robustness of our results. In the Marked-up manuscript, modifications suggested by reviewers are highlighted in yellow.

Reviewer #1: Very interesting, clinically relevant and topical Digital Twin Approach for Simultaneous Reconstruction of Brain Anatomy and Dynamics from Neural Data. I very much enjoyed reviewing this paper and think it could be of value to others.

We are extremely grateful to the Reviewer for the inspiring and gratifying comments about our work.

Reviewer #2: The manuscript presents an ambitious and technically impressive end-to-end pipeline (FEDE) integrating MRI-derived anatomy (including tractography, conduction velocity estimation, and FEM forward modeling) with whole-brain neural mass simulations to reproduce subject EEG. The work is timely and could be impactful as a methods contribution. However, several issues limit interpretability and rigor of the current evidence.

We thank the Reviewer for these kind words and inspiring suggestions. Please, find below our replies. In the Marked-up version of the manuscript, modifications are reported highlighted in yellow.

First, conclusions about ASD-related “aberrant synaptic transmission” are not supported by a single-case fit without uncertainty quantification; please reframe as feasibility/hypothesis-generating or add controls/replication.

We thank the Reviewer for this important comment that helped us to clarify the aim of our work.

We agree that conclusions regarding ASD-related “aberrant synaptic transmission” cannot be firmly supported by a single-case model fit without uncertainty quantification or comparison with a control cohort. The primary aim of the present work was indeed to demonstrate the feasibility of the proposed modeling framework rather than to draw definitive pathophysiological conclusions.

As correctly pointed out by the reviewer, some wording in the Abstract and Author Summary could nevertheless be interpreted as overly strong. We have therefore revised these sections to explicitly present the findings as preliminary and exploratory.

The Abstract sentence:

*“FEDE predicted patient-specific anomalies in synaptic transmission, coherently with ASD pathophysiology.”*

Has been changed to:

“*FEDE estimated possible patient-specific anomalies in synaptic transmission, coherently with ASD pathophysiology.”*

Similarly, the Author Summary sentence:

*“We applied our approach to a young child with ASD, accurately reproducing their recorded brain activity and revealing individualized alterations in synaptic transmission consistent with known ASD mechanisms.”*

Has been changed to:

*“We applied our approach to a young child with ASD, accurately reproducing their recorded brain activity and suggesting possible individualized alterations in synaptic transmission consistent with known ASD mechanisms.”*

Furthermore, in the *“FEDE employs Hierarchical Parameter Optimization to reconstruct personalized multi-scale structural features of the patient’s brain” paragraph* of the Results section, the sentence:

*“Since model parameters describe structural features spanning multiple scales, the optimal values identified by FEDE can be compared with physiological values to support diagnosis of pathological conditions.”*

Has been updated to:

*“Since model parameters describe structural features spanning multiple scales, the optimal values identified by FEDE can be compared with physiological values to make hypotheses on underlying patient condition.”*

We believe that these amendments better reflect the purely inferential link between quantities identified with the SHAP analysis and underlying pathophysiological mechanism. Future work will extend this framework to larger cohorts including typically developing (TD) controls, which will allow a more rigorous evaluation of potential ASD-related alterations and enable statistical comparisons across subjects.

Second, the optimization and evaluation metrics need clearer, reproducible definitions: Eq. (4) (FitPSD) is not a well-specified scalar objective as written, and the combined score formulation should be detailed (averaging across channels/frequencies, use of absolute/squared error, etc.).

We thank the Reviewer for helping us improve the clarity in the definition of Eq. (4).

We agree that the original formulation of Eq. (4) (FitPSD) did not sufficiently specify how the objective function was computed. In the revised manuscript we have now updated the equation, also clarifying the definition of this metric:

*“*$Fit PSD=1-MAE[{log}_{10}\left( {PSD}_{exp})-{log}_{10}\left( {PSD}_{sim} \right) \right] (4)$

*Where* $Corr$ *is the standard Pearson correlation. In the* $Fit PSD$*function, the PSD were truncated at 20 Hz (this was done to improve the efficacy of the loss function in locating the best fits), MAE is the Mean Absolute Error as implemented by sklearn.metrics, PSD values were averaged among channels. The best match between simulated and actual EEG was computed using these metrics as the combination of model parameters that maximized the* $\sqrt{{Fit PSD}^{2}+ {Fit FC}^{2}}$ *sum.”*

In particular, the revised description now specifies how the mismatch between empirical and simulated power spectral densities is computed across frequencies and channels, including the averaging procedure and the error metric used.

These changes were introduced to ensure that the optimization and evaluation metrics are fully reproducible. The corrected equation and the corresponding methodological description are now reported in the Methods section.

Third, key statistical comparisons treat massively correlated samples (matrix entries/vertices) as independent, inflating significance; report effect sizes and use dependence-aware inference where possible.

We thank the Reviewer for raising this relevant methodological concern, which led to further analysis increasing the robustness of our results. We agree that treating individual entries of connectivity matrices or spectral vectors as independent observations can artificially inflate statistical significance due to strong dependencies among elements.

To address this issue, we revised the statistical analysis using a dependence-aware approach:

1. For functional connectivity matrices, we have now restricted the analysis to the upper triangular part of the matrices to avoid redundancy due to matrix symmetry.
2. Instead of relying on parametric tests assuming independence, we implemented a non-parametric permutation test to assess statistical differences between simulated and empirical measures.
3. We additionally report effect sizes (Cohen’s d) to quantify the magnitude of differences between empirical quantities and their simulated counterparts.

Using this approach, we obtained the following results:

- Power spectral density: d = −0.034, permutation p = 0.39
- Functional connectivity: d = 0.025, permutation p = 0.85

Both effect sizes are extremely small (|d| < 0.05), indicating negligible differences between simulated and empirical distributions, and permutation tests confirmed the absence of statistically significant differences.

These results support the conclusion that the simulations reproduce the empirical spectral and connectivity properties without detectable bias. This analysis has been added to the "*FEDE replicates accurately the spatiotemporal structure of experimental EEG recordings*" paragraph of the Results section, reading:

*“Since both PSD and FC values represent data that are highly intercorrelated, to better quantify the similarity of FEDE-derived metrics with their empirical counterparts, a dependence-aware statistical comparisons was conducted between empirical and simulated PSD and FC matrices. This analysis revealed negligible differences for both features, with extremely small effect sizes and non-significant permutation tests (PSD: Cohen’s d = −0.034, p = 0.39; FC: Cohen’s d = 0.025, p = 0.85), indicating that the simulations closely reproduce the empirical distributions..”*

The novel statistical analysis in now explained in the *“Statistical Analysis”* paragraph of the Methods section, reading:

*“To compare empirical and simulated data we also performed dependence-aware statistical comparisons between simulated and empirical measures. For functional connectivity matrices, analyses were restricted to the upper triangular part of the matrices to avoid redundancy due to symmetry. Statistical differences were assessed using non-parametric permutation tests, which do not assume independence between observations. In addition to p-values, we quantified the magnitude of differences using Cohen’s d effect sizes.”*

Fourth, FC based on Pearson correlation remains vulnerable to volume conduction; make leakage-robust metrics (e.g., PSI/wPLI/imaginary coherence) primary or report robustness.

We thank the Reviewer for this important comment.

In this study, Pearson correlation was initially used as the primary functional connectivity metric because our modeling framework aims to jointly reproduce both the anatomical structure and the resulting functional interactions. Pearson correlation is sensitive to shared signal components and therefore reflects both neural dynamics and the anatomical constraints imposed by the structural connectome. In the context of the present modeling approach, this property allows us to evaluate whether the simulated activity reproduces the empirical large-scale functional patterns generated by the underlying anatomy.

We acknowledge that Pearson correlation can be affected by volume conduction. To address this concern and assess the robustness of the results, we additionally evaluated functional connectivity using a leakage-robust metric, namely the phase slope index (PSI). This analysis yielded similar results (r = 0.53, p < 0.00001) even without performing a dedicated parameter refitting. The results are now reported in the “*FEDE accurately replicates experimental EEG dynamics across multiple domains*” paragraph of the Results section, reading:

*“FC replication was also computed with phase slope index (PSI)* (1)*, which is less affected by volume conduction, obtaining similar results (r=0.53, p<0.00001) without a dedicated parameter fitting (which would have likely increased the fit value for the PSI metric).”*

The PSI computation has also been added to the “*EEG features: functional connectivity*” paragraph of the Methods section, which has been improved to better clarify the choice of Pearson correlation as primary FC metric. The novel version of the paragraph reads:

*“****EEG features: functional connectivity***

*Functional connectivity was computed with standard Pearson correlation, considering only significant (p<0.05, Bonferroni correction) values. The choice of the Pearson correlation metric was motivated by the limited number of channels, reducing volume conduction effects* (1)*. Furthermore, volume conduction is heavily determined by the anatomical structure* (2)*, meaning that the correct replication of Pearson correlation requires to capture both the neural activity and the underlying anatomy at once. Nonetheless, other FC metrics were also tested, computing Phase Slope Index in both empirical and simulated data, with similar results with respect to those obtained for Pearson correlation.”*

Finally, an ablation study isolating the contribution of FEM vs BEM, personalized CV, cortical resolution, and local connectivity would strengthen causal attribution of performance gains.

We thank the Reviewer for this valuable suggestion, which led to a relevant addition to our work.

To clarify the contribution of the main components of the modeling framework, we performed an ablation-style comparison across key methodological choices, including the type of lead-field model (BEM vs FEM), the representation of conduction velocity (constant vs spatially mapped), and the spatial scale of simulations (region-based vs surface-based simulations).

The results of this comparison are now reported in Supplementary Table S3, which summarizes the fitting performance for both functional connectivity (Fit FC) and power spectral density (Fit PSD) across the different modeling variants:

| ***Variant*** | ***LFM Model*** | ***CV Model*** | ***Simulation Scale*** | ***Fit FC*** | ***Fit PSD*** |
| --- | --- | --- | --- | --- | --- |
| *01* | *BEM* | *Const* | *Region* | *0.676* | *0.8429* |
| *02* | *FEM* | *Const* | *Region* | *0.7742* | *0.8398* |
| *03* | *BEM* | *Map* | *Region* | *0.6741* | *0.8465* |
| *04* | *FEM* | *Map* | *Region* | *0.7729* | *0.8419* |
| *05* | *BEM* | *Const* | *Surface* | *0.6975* | *0.8773* |
| *06* | *FEM* | *Const* | *Surface* | *0.8041* | *0.8681* |
| *07* | *BEM* | *Map* | *Surface* | *0.6977* | *0.8773* |
| *08* | *FEM* | *Map* | *Surface* | *0.8041* | *0.8683* |

***Table S3.***

***Comparison between simulated PSDs and FCs depending on simulation features.*** *Quality of fit for functional connectivity (Fit FC, fifth column) and PDS (Fit PSD, sixth column) using simulations with finite or boundary element (FEM or BEM) lead field matrix (LFM, second column), Constant or map-based Conduction Velocity (CV, third column), and region based vs surface based simulation scale (fourth column). Simulations have been made using the optimal combination of model parameter (see Table S2).*

The full ablation comparison is now reported in the “*FEDE accurately replicates experimental EEG dynamics across multiple domains*” paragraph of the Results section, reading:

*“To quantify the relative influence of key modeling assumptions on the performance metrics, we performed an ablation study (Fig 5E)  in which we systematically varied the type of lead field model (BEM vs FEM), the formulation of conduction velocity (constant vs subject-specific spatial map), and the simulation scale (region-based vs surface-based). For each combination, we computed the resulting Fit PSD and Fit FC values. We then carried out a sensitivity analysis to determine which assumptions had the largest impact on these metrics. This analysis revealed that the simulation scale (region vs surface) exerted the strongest influence on spectral fit (ΔFit PSD = 0.029975), whereas the lead field model (BEM vs FEM) had the largest impact on functional connectivity fit (ΔFit FC = 0.102500). In contrast, the conduction velocity model (constant vs spatial map) had only negligible effects on both metrics, indicating that Fit PSD and Fit FC are largely insensitive to this assumption.”*

Figure 5 has also been updated according to the new Results. We hereby report the new version:

***Figure 5: FEDE significantly outperforms standard digital twin models in replicating experimental recordings. (A):*** *Comparison of FC matrices obtained from experimental recordings (left), simulated EEG using standard model (middle-left) and simulated EEG using FEDE (middle-right).* *FC matrices computed from standard methodology showed different patterns with respect to those observed in experimental FC. Standard model led to simulated EEG with a smaller Pearson r-value with experimental FC compared to the value obtained with FEDE (right).* ***(B):*** *PSD computed from standard model simulations (left) and from FEDE simulations (middle). The EEG simulated with the standard model presented reduced similarity with experimental recordings, with different dominant frequency and no second peak in low-alpha band. This caused the standard model simulation to have a smaller Pearson r-value with experimental PSD compared to the value obtained with FEDE (right).* ***(C):*** *Fit values between experimental and simulated FC for 100 best standard model and FEDE simulations. FEDE presented higher fit values in almost all simulations.* ***(D):*** *Fit values between experimental and simulated PSD for 100 best standard model and FEDE simulations. FEDE presented higher fit values with high significance.* ***(E):*** *Sensitivity analysis reporting the relationship between modelling choices and Fit PSD and Fit FC values. Modeling choices include: (i) lead field model (boundary element method, BEM, vs finite element method, FEM), (ii) conduction velocity model (spatially constant vs personalized map), and (iii) simulation scale (region-based vs surface-based simulations). Significance notation: **** stands for p<0.00001.*

Overall, the results indicate that the largest performance differences are associated with the choice of the lead-field model and the simulation resolution, whereas the representation of conduction velocity had only a minor effect for the examined parameter configuration. These results support the added value of the proposed FEDE pipeline compared with more conventional modeling configurations.

This analysis is based on a methodological process now reported in the new Methods section:

*“****Ablation study and sensitivity analysis***

*To quantify the influence of key modeling assumptions on model performance, we conducted an ablation study in which three methodological components were systematically varied: (i) the lead field formulation (boundary element method, BEM, vs finite element method, FEM), (ii) the conduction velocity model (spatially constant vs personalized map), and (iii) the simulation scale (region-based vs surface-based simulations). All possible combinations of these assumptions were evaluated, resulting in eight simulation configurations. For each configuration, we computed two performance metrics quantifying the agreement between simulated and empirical data: (Fit PSD and Fit FC). To assess the relative impact of each modeling assumption, we performed a sensitivity analysis by computing, for each factor, the absolute difference between the average metric values obtained across its levels (e.g., FEM vs BEM). This effect magnitude (Δ metric) provides a direct estimate of how strongly each modeling choice influences the resulting model fit.”*

Reviewer #3: This is an excellent and technically sophisticated study. Although it is based on a single-case design, the authors have appropriately acknowledged this as a limitation. However, several aspects of the Methods section would benefit from further clarification:

We are grateful to the Reviewer for the inspiring and thoughtful comments. Please, find below our point-by-point reply, while modifications in the marked-up manuscript are highlighted in yellow.

The diffusion protocol (single-shell, 30 directions, single b-value) may limit the reliability of constrained spherical deconvolution, tractography, and derived structural connectivity measures. The authors are requested to provide additional explanation or justification regarding how these limitations were addressed or mitigated.

We thank the Reviewer for this important comment that helped us clarify our methodology.

While multi-shell acquisitions with a larger number of directions would provide additional microstructural information, the adopted SS3T-CSD framework has been specifically developed to allow robust estimation of fiber orientation distributions and tractography from single-shell diffusion MRI data. We have clarified these methodological aspects in the revised manuscript. Addition have been made in the “*Constrained spherical deconvolution*” paragraph of the Methods section, reading:

*“Due to the limited diffusion data available (only one gradient valuewith B=1000s/mm²), the response functions were generated using MRtrix3Tissue (*[*https://3Tissue.github.io*](https://3tissue.github.io/)*), a fork of MRtrix3* (3)*. This latter implements the single-shell three-tissue constrained spherical deconvolution (SS3T-CSD) approach* (4) *. This method enables the estimation of tissue-specific fiber orientation distributions (FODs) for white matter (WM), grey matter (GM), and cerebrospinal fluid (CSF) even from single-shell diffusion data. The algorithm relies on an iterative estimation of tissue response functions and spatial constraints to separate anisotropic WM signal from isotropic GM and CSF components. SS3T-CSD has been widely adopted in the literature and successfully applied to single-shell datasets (e.g., Newman et al.* (5)*; Yang et al.* (6)*, Jaroszynski et al.* (7)*).*

*Regarding the relatively limited number of diffusion directions (30), preprocessing was performed using the dwifslpreproc pipeline in MRtrix3, which internally calls FSL eddy for correction of motion and eddy-current distortions. In accordance with the FSL recommendations for datasets with fewer than 60 diffusion directions, the option --slm=linear was used during eddy correction, which improves the robustness of the estimation in low-direction acquisitions. The --slm=linear option constrains the eddy-current–induced field to a linear spatial model, reducing the number of parameters to be estimated during eddy correction. This simplification improves the stability and robustness of the correction when diffusion datasets contain a limited number of gradient directions, as in the present acquisition. This approach is also described in the methodological work by Andersson & Sotiropoulos* (8) *and Andersson et al.* (9)*.“*

The use of synthetic reverse phase encoding and the estimation of anisotropic conductivity require stronger methodological justification and, where possible, supporting validation.

We thank the Reviewer for raising this methodological point.

While both synthetic reverse phase encoding and the estimation of anisotropic conductivity rely on model-based estimations, they represent well-established solutions when direct measurements (e.g., reversed phase encoding acquisitions or direct conductivity measurements) are not available. We have clarified these methodological choices and added the relevant references in the revised manuscript.

The novel methodological references for the reverse phase encoding are reported in the “*MRI Preprocessing*” paragraph of the Methods section, reading:

*“For susceptibility distortion correction, a synthetic reverse phase–encoding (RPE) image was generated using SynB0-DISCO (https://github.com/MASILab/Synb0-DISCO), following the method described by Schilling et al. (10,56). This approach uses a deep learning model trained on paired T1-weighted and reversed phase–encoded diffusion images to synthesize a distortion-free b0 image, which can then be used within standard FSL topup/eddy pipelines. SynB0-DISCO was specifically developed to enable susceptibility distortion correction when reversed phase–encoding images are not available and has been validated against acquisitions with real reversed phase–encoding data. The method has been increasingly adopted in diffusion MRI studies where fieldmap or reverse PE data are unavailable.”*

Regarding the estimation of anisotropic conductivity, this has now been clarified in the novel version of the “Reconstruction of biophysical 3D Head Model and Calculation of Lead-Field Matrix” paragraph of the Methods section, reading:

*“In order to calculate the lead-field matrix, we first reconstructed a biophysical 3D head model of the patient with a 12 tissues segmentation using SimNIBS4.1.0. The T1-w and coregistered/regridded T2-w were used as input to charm command together with the Freesurfer anatomical reconstruction folder (the SimNIBS template was coregistered to the T1-w using Freesurfer to find the Transformation matrix used to initialize the affine registration of the SimNIBS template to the subject MR). Then also the DWI image was imported with the dwi2cond command (based on FSL dtifit), in order to calculate the tensors necessary to compute the anisotropic properties of conductivity for GM and WM (see Supplementary Figure S5) using the “Volume Normalized” algorithm as shown by Opitz et al (15). This approach estimates conductivity tensors from diffusion tensor imaging by assuming a proportional relationship between water diffusion and electrical conductivity in white matter. The method relies on diffusion tensor estimation (FSL dtifit) followed by tensor-to-conductivity conversion as described in the SimNIBS framework. This procedure has been widely used for constructing subject-specific conductivity models for electric field simulations in transcranial stimulation and related modeling studies.”*

Clarification is needed regarding the apparent discrepancy between the empirically recorded EEG channels (21 channels, reduced to 16 after preprocessing) and the 64-channel forward model used for simulations.

We thank the Reviewer for requesting clarification on this point, which was not properly addressed in the previous version of the manuscript.

The 64-channel forward model was chosen since it presented the same 10-10 outline adopted by the empirical cap. This allowed us to select all empirical channels as a subset of the virtual cap used in the simulation, while also allowing for flexibility due to the presence of extra channels (that could be used also in combination with higher density caps).

We have clarified this procedure in the revised manuscript to avoid any ambiguity regarding the relationship between the empirical EEG montage and the forward model used for simulations. These additions have been made to the “*Reconstruction of biophysical 3D Head Model and Calculation of Lead-Field Matrix*” paragraph of the Methods section, reading:

*“The empirical EEG recordings were acquired using a 21-channel cap, which resulted in 16 channels after preprocessing. For the forward model used in the simulations, a standard 64-channel montage following the international 10-10 system was initially employed to construct the leadfield matrix. This choice provides a denser spatial sampling of the scalp and improves the numerical stability and spatial representation of the forward model.*

*Because both the empirical montage and the 64-channel template follow the 10-10 coordinate system, the electrode positions corresponding to the recorded channels directly overlap with their counterparts in the 64-channel configuration. Therefore, for the comparison with the empirical EEG signals, only the subset of electrodes corresponding to the 16 recorded channels was retained, while the remaining channels of the 64-channel template were excluded from the analysis.”*

A sensitivity analysis examining the robustness of the parameter optimization procedure and key modeling assumptions would strengthen confidence in the reported conclusions.

We thank the Reviewer for this suggestion.

A sensitivity analysis of the modeling framework was performed through two complementary approaches. First, we applied SHAP analysis (see Figure 6D) to quantify the contribution of individual parameters to the optimization results. This approach allowed us to evaluate the relative importance of the model parameters and to assess the robustness of the optimization procedure with respect to variations in the parameter space.

Following the Reviewer’s suggestion, we also performed an additional analysis to examine the robustness of the results with respect to key modeling assumptions, which was requested also by Reviewer 1. Specifically, we compared different configurations of the modeling framework, including the type of lead-field model (BEM vs FEM), conduction velocity representation (constant vs spatially mapped), and simulation domain (regional vs surface-based simulations). The results of this comparison are reported in the “*FEDE accurately replicates experimental EEG dynamics across multiple domains*” paragraph of the Results section, also including an updated version of Figure 5 to present the novel analysis. Numerical values are also reported in Table S3, which summarizes the fitting performance for both functional connectivity (Fit FC) and power spectral density (Fit PSD) across these alternative configurations.

Overall, the results indicate that the main conclusions of the study remain stable across the tested modeling variants. In particular, the choice of conduction velocity representation had only a minor effect on the fitting metrics for the examined parameter configuration, while the differences between BEM and FEM lead-field models did not substantially alter the observed patterns. Please see also the reply to the last point (point number 5) of Reviewer 2 for reference.

References

1.	Bastos AM, Schoffelen JM. A Tutorial Review of Functional Connectivity Analysis Methods and Their Interpretational Pitfalls. Front Syst Neurosci. 2016 Jan 8;9. doi:10.3389/fnsys.2015.00175

2.	van den Broek SP, Reinders F, Donderwinkel M, Peters M. Volume conduction effects in EEG and MEG. Electroencephalography and clinical neurophysiology. 1998;106(6):522–34.

3.	Tournier JD, Smith R, Raffelt D, Tabbara R, Dhollander T, Pietsch M, et al. *MRtrix3*: A fast, flexible and open software framework for medical image processing and visualisation. NeuroImage. 2019 Nov 15;202:116137. doi:10.1016/j.neuroimage.2019.116137

4.	Dhollander T, Connelly A. A novel iterative approach to reap the benefits of multi-tissue CSD from just single-shell (+ b= 0) diffusion MRI data. In: proc ISMRM [Internet]. 2016 [cited 2026 Mar 9]. p. 3010. Available from: https://www.researchgate.net/profile/Thijs-Dhollander/publication/301766619_A_novel_iterative_approach_to_reap_the_benefits_of_multi-tissue_CSD_from_just_single-shell_b0_diffusion_MRI_data/links/5726b0e608aee491cb3f111d/A-novel-iterative-approach-to-reap-the-benefits-of-multi-tissue-CSD-from-just-single-shell-b0-diffusion-MRI-data.pdf

5.	Newman BT, Dhollander T, Reynier KA, Panzer MB, Druzgal TJ. Test–retest reliability and long-term stability of three-tissue constrained spherical deconvolution methods for analyzing diffusion MRI data. Magnetic Resonance in Medicine. 2020;84(4):2161–73. doi:10.1002/mrm.28242

6.	Yang JYM, Chen J, Alexander B, Schilling K, Kean M, Wray A, et al. Assessment of intraoperative diffusion EPI distortion and its impact on estimation of supratentorial white matter tract positions in pediatric epilepsy surgery. Neuroimage Clin. 2022;35:103097. doi:10.1016/j.nicl.2022.103097 PubMed PMID: 35759887; PubMed Central PMCID: PMC9250069.

7.	Jaroszynski C, Attyé A, Job A, Delon-Martin C. Tracking white-matter brain modifications in chronic non-bothersome acoustic trauma tinnitus. Neuroimage Clin. 2021;31:102696. doi:10.1016/j.nicl.2021.102696 PubMed PMID: 34029920; PubMed Central PMCID: PMC8163994.

8.	Andersson JLR, Sotiropoulos SN. An integrated approach to correction for off-resonance effects and subject movement in diffusion MR imaging. Neuroimage. 2016 Jan 15;125:1063–78. doi:10.1016/j.neuroimage.2015.10.019 PubMed PMID: 26481672; PubMed Central PMCID: PMC4692656.

9.	Andersson JLR, Graham MS, Zsoldos E, Sotiropoulos SN. Incorporating outlier detection and replacement into a non-parametric framework for movement and distortion correction of diffusion MR images. Neuroimage. 2016 Nov 1;141:556–72. doi:10.1016/j.neuroimage.2016.06.058 PubMed PMID: 27393418.
